# Antimicrobial and anti-biofilm activities of *Vernonia amygdalina* and *Senna siamea* leaf extracts against pathogenic bacteria

**DOI:** 10.1371/journal.pone.0358840

**Published:** 2026-10-01

**Authors:** Sumaila Mohammed, Zakari Abdul-Karim, Emmanuel Ndezure, Matthew Glover Addo, Kwadwo Boampong

**Affiliations:** Department of Theoretical and Applied Biology, KNUST, Kumasi, Ghana; Government College University Faisalabad, PAKISTAN

## Abstract

The study assessed the antibacterial and antibiofilm capability of *Vernonia amygdalina* and *Senna siamea* against both ESBL- and non-ESBL-producing bacteria. The result from the phytochemical screening revealed that secondary metabolites such as alkaloids, flavonoids, saponins, tannins, coumarins, triterpenoids, and phytosteroids were present in both plants, except glycosides, which were exclusively detected in *Vernonia amygdalina*. The antibacterial activity showed that *Senna siamea* had its highest zone of inhibition against *K. pneumoniae*, whereas *Vernonia amygdalina* recorded its highest effect on *E. coli*. The MIC values for *Senna siamea* ranged from 6.25 to 25.00 mg/mL, while the MICs of *Vernonia amygdalina* ranged from 12.50 ± 0.00 to 50.00 ± 0.00 mg/mL. Combination testing indicated synergy between the two extracts only for *E. coli*. Antibiofilm assays showed clear dose-dependent inhibition. At 100 mg/mL, *Vernonia amygdalina* showed 99.01% inhibition against ESBL *K. pneumoniae*, while *Senna siamea* at the same concentration reached 99.72% against *S. aureus*. These data demonstrate the relevant antibacterial and antibiofilm activities for each extract and support continuing efforts that could include them in new strategies against resistant pathogens, including ESBL-producers.

## Introduction

Pathogenic bacteria pose a global health threat, complicating infection treatment, raising healthcare costs, and directly causing about 9% of deaths worldwide each year [1,2]. Resistance mechanisms in bacteria continue to increase, leading to the rise of multidrug-resistant (MDR) strains despite the development of antibiotics [3]. In 2019, there were 13.7 million deaths related to infections [4], with 1.27 million and 4.95 million deaths linked to antimicrobial resistance (AMR), both directly and indirectly [5]. Death rates associated with AMR vary significantly by region; for example, Australasia had an AMR-related mortality rate of 28.0 per 100,000, while western sub-Saharan Africa’s rate was 114.8 per 100,000 [2]. AMR also has economic impacts, likely reducing global GDP growth by 3.8%, with total economic losses projected to exceed US$100 trillion by 2050, especially threatening lower-middle-income countries [5]. The worldwide burden of antimicrobial resistance stems from the misuse and overuse of antibiotics, along with social and economic factors [6]. Moreover, globalization related to trade and commerce accelerates the spread of resistant bacteria across borders [6].

Extended-spectrum beta-lactamase is one of the major resistance factors of pathogenic bacteria. Beta-lactam antibiotics, such as penicillins, cephalosporins, and aztreonam, are degraded by ESBL enzymes [7]. ESBL-producing enterobacteriaceae, such as *Escherichia* and *Klebsiella pneumoniae*, cause healthcare-associated infections [8]. This is one of the factors limiting treatment options and increasing the risk of treatment failure. The other major factor of bacterial persistence is biofilm production. Biofilms are microbial communities with a complex structure, encapsulated in an extracellular matrix of bacterial origin [9]. Biofilms attach to living or non-living surfaces. Biofilms protect bacterial cells from the host immune response and antimicrobial agents [9].

The medicinal plants have continued to attract attention as alternative sources of antimicrobial agents. The traditional medicine systems have widely used plant extracts to control microbial infections due to the variety of bioactive compounds found in the plants. *Senna siamea* and *Vernonia amygdalina* are among the medicinal plants that have been widely used in African traditional medicine to control various infections, including microbial infections [10,11]. The plants have been found to contain several phytochemicals, such as alkaloids, flavonoids, tannins, and saponins, and all of these have been found to possess antimicrobial activity against several pathogenic microorganisms [12,13]. Earlier studies have documented the antibacterial activity of the plant extracts against several bacterial species. The ethnomedicinal value of the plants, their phytochemical composition, and their antimicrobial activity were the reasons why the plants were selected for the study.

Most studies have centered on the general antimicrobial activity of the plants. Few studies have evaluated the activity of the plants against ESBL-producing bacteria and non-ESBL-producing bacteria. Information on the activity of the plants against the inhibition of bacterial biofilm formation is also limited. Therefore, this study aims to further evaluate the antimicrobial activity of *Senna siamea* and *Vernonia amygdalina* against ESBL-producing bacteria and non-ESBL-producing bacteria, as well as their activity against the inhibition of bacterial biofilm formation. Such information would also give more insights into the possible use of the plants in addressing infections caused by resistant and nonresistant bacterial strains.

## Materials and methods

### Test organisms and inoculum standardization

Typed laboratory strains of the test organisms have been used for the study. These test organisms include *Salmonella typhi*, *Staphylococcus aureus*, *Escherichia coli*, beta lactamase producing *Escherichia coli* (*E. coli* ESBL), *Klebsiella pneumoniae*, and extended spectrum beta lactamase producing *Klebsiella pneumoniae* (*K. pneumoniae* ESBL). These microorganisms were obtained from the Pharmaceutical Microbiology Laboratory, Department of Pharmaceutics, Faculty of Pharmacy and Pharmaceutical Sciences, Kwame Nkrumah University of Science and Technology, Kumasi, Ghana. These microorganisms were used in this study since they are maintained as laboratory stock cultures.

*Escherichia coli* ATCC 25922 was used as a reference strain in this study. The reference strain was used to ensure the reliability of the results obtained from the antimicrobial activity tests.

The bacteria used in this study were chosen because they are common pathogenic bacteria that cause infections and antimicrobial resistances in humans. *Escherichia coli* and *Klebsiella pneumoniae* were chosen because they are common pathogenic bacteria that cause infections and antimicrobial resistances in humans, particularly the extended-spectrum beta-lactamase resistances. *Salmonella typhi* was chosen because it is a Gram-negative pathogenic bacterium that causes typhoid fever in humans. *Staphylococcus aureus* was chosen because it is a Gram-positive pathogenic bacterium that causes infections in the skin, wounds, and other body systems in humans. The choice of Gram-negative and Gram-positive bacteria was chosen to assess the antimicrobial effects of the plant extracts on different types of bacteria.

The bacterial suspensions were prepared using the bacteria cultured in the lab and standardized to a density of 1.0 x 10⁸ CFU/mL, which is equivalent to the 0.5 McFarland standard.

### Collection, identification, and preparation of plant samples

The leaves of *Senna siamea* and *V. amygdalina* were collected from the KNUST horticulture garden, with the following Global Positioning System (GPS) coordinates: longitude: 6.678996, latitude: - 1.56569. Permission to conduct the study and to collect plant samples was obtained from the Department of Theoretical and Applied Biology and the Department of Horticulture, Kwame Nkrumah University of Science and Technology (KNUST), Kumasi, Ghana, which manages the horticulture garden where the samples were collected. No formal written permit or permit number was required for this study because the plant species collected are common, non-endangered, non-protected species widely cultivated in Ghana. Also, the collection was conducted on the university-owned land for academic research purposes, and access was granted directly by the responsible department. In the horticulture lab, the leaves were oven-dried for 24 hours at 45°C and were finely ground into powder. Identification and authentication were done at the Department of Herbal Medicine, Faculty of Pharmacy and Pharmaceutical Sciences, KNUST, Kumasi, Ghana.

### Extraction of the plant samples

Leaves of *Senna siamea* and *Vernonia amygdalina* washed with distilled water, dried at room temperature (25-28°C) in the shade, and left to dry for 10 days to prevent the degradation of bioactive compounds. The leaves were milled into a fine powder using a grinder.

Fifty grams of each milled leaf material were measured and transferred into a separate sterile conical flask. The leaf material was soaked in 70% ethanol, extracted using a cold maceration technique, left to soak for 72 hours, and intermittently shaken once a day to aid the solvent in penetrating the leaf material, thereby extracting phytochemicals from the leaf material. After maceration, the mixture was filtered using sterile white muslin cloth, followed by filter paper, No. 1, to obtain a clear solution.

The clear solution was concentrated using a rotary evaporator at 40°C to separate the solvent, ethanol, from the solution. The concentrated solution was left to dry using a water bath at a temperature of 40°C to obtain a semi-solid crude solution. Percentage extraction yield was calculated using the formula:


$$\begin{array}{c} \text{Extraction yield percent}=(\text{weight of dried extract}÷\text{weight of initial plant powder})\times \text{100}. \end{array}$$

The crude extracts were sterilized using membrane filtration with a 0.22 µm sterile syringe filter. The extracts were then placed in a sterile McCartney bottle and stored at 4°C prior to the antimicrobial bioassays.

### Preparation of stock and working solutions

A stock solution of the individual crude extracts was prepared by dissolving a known weight of the dried extracts in sterile distilled water with 5 percent dimethyl sulfoxide, which is used to increase the solubility of the extracts. The stock solution was prepared at a concentration of 100 mg/mL. A series of twofold dilutions was carried out using the stock solution and sterile Mueller Hinton broth to obtain the required working concentrations [14].

### Qualitative and quantitative phytochemical screening

*Senna siamea* and *Vernonia amygdalina* ethanolic extracts were tested for qualitative and quantitative phytochemical composition to determine the existence of essential bioactive compounds, including alkaloids, saponins, tannins, flavonoids, glycosides, coumarins, phenols, steroids, and triterpenoids, using standard procedures documented by [15]. Quantitative phytochemical values are expressed as mg per gram of the dried crude extract.

### Agar well diffusion assay

Antibacterial activity of the plant extracts was carried out using the agar well diffusion method. Sterile Mueller Hinton agar medium, 20 ml, was poured into sterile 90 mm diameter petri plates and allowed to solidify under aseptic conditions. Fresh bacterial cultures were prepared and standardized to match the 0.5 McFarland turbidity standard, which is equivalent to about 1.0 x 10⁸ CFU per ml.

The standardized bacterial cultures were evenly spread over the Mueller-Hinton agar plates using sterile cotton swabs to obtain uniform bacterial lawns. The plates were allowed to air-dry at room temperature for 15 minutes to enable the absorption of the inoculum into the agar surface.

A total of four wells were aseptically prepared in the agar plates using a sterile cork borer with a diameter of 6 mm. A volume of 100 microliters of the plant extracts was added to the wells at concentrations of 25, 50, 100, and 200 mg per ml prepared in dimethyl sulfoxide with the final concentration not exceeding 1 percent.

Ciprofloxacin at a concentration of 50 µg of ciprofloxacin per mL of medium served as the positive control for the antibacterial activity of the assay system. One percent dimethyl sulfoxide served as the negative control for the antibacterial activity of the assay system.

The plates were kept at 4°C for 1 hour to allow for the diffusion of the extracts into the agar medium. The plates were then incubated aerobically at 37°C for 18 to 24 hours. After the incubation period, the diameters of the zones of inhibition were determined in millimeters using a Vernier caliper. The results were obtained in triplicate and are expressed as mean ± standard deviation.

### Minimum inhibitory concentration (MIC) determination

The minimum inhibitory concentration of the plant extracts was determined using the broth dilution method. This method involves the preparation of serial twofold dilutions of the plant extracts in a sterile nutrient broth, which provides different concentrations of the extract. One milliliter of the nutrient broth was transferred into a series of sterile test tubes, followed by the addition of one milliliter of the plant extract, which was then serially diluted to achieve the required concentrations.

A standardized bacterial inoculum, corresponding to a 0.5 McFarland standard, which represents a bacterial density of approximately 1.0 x 10^8 CFU per mL, was prepared from fresh bacterial cultures of each of the test organisms. From this suspension, 0.1 mL of the bacterial inoculum was transferred into each of the tubes containing the mixture of the extract and nutrient broth.

All the tubes were incubated at 37°C for 24 hours. After the incubation period, the tubes were checked for bacterial growth based on turbidity. The MIC was recorded as the minimum concentration of the extract from which there was no visible bacterial growth compared to the growth control [16].

### Minimum bactericidal concentration (MBC) determination

The minimum bactericidal concentration was determined from the tubes that showed no visible growth during the MIC determination. A loopful from each of the tubes was aseptically subcultured onto sterile nutrient agar plates using the spread-plate method. The plates were incubated at 37°C for 24 hours.

After the incubation period, the plates were checked for bacterial growth. The minimum concentration from which there was no bacterial growth on the agar plates was recorded as the minimum bactericidal concentration.

After incubation, the plates were observed for signs of bacterial growth. The highest concentration of the extract from which no bacteria were observed on the agar plates was taken as the minimum bactericidal concentration [12].

### Synergistic interaction analysis using the checkerboard microdilution method

The checker board micro dilution method was used to assess the interaction between *Senna siamea* and *Vernonia amygdalina* extracts and their effect on the test organisms using previously described methods. The assay assesses the combined inhibitory effect of two agents on microbial growth and their interaction to be synergistic, additive, indifferent, or antagonistic.

Serial two-fold dilutions of one extract were prepared on the horizontal axis of sterile 96 well microtiter plates, and serial dilutions of the second extract were prepared on the vertical axis of the same plates. These dilutions combined to create different combinations of concentrations of the two extracts in each well of the microtiter plate. The concentrations of each extract used in this study ranged from 0.78 to 100 mg per mL. These concentrations were chosen based on preliminary antimicrobial screening and the observed minimum inhibitory concentration to detect any interaction effects at inhibitory concentrations.

Sterility control wells contained sterile broth without bacterial inoculum to check the sterility of the medium, and the growth control wells contained bacterial inoculum with broth without plant extract to confirm the viability of the bacteria during the experiment.

The microtiter plates were incubated aerobically at 37°C for 18 to 24 hours. After the incubation period, the bacteria l growth was determined by measuring the optical density using a microplate reader (Synergy H1, BioTek Instruments, USA) at a wavelength of 590 nm. The optical density indicated the extent of bacterial growth in each microtiter well. The lower the optical density values, the higher the inhibition of bacterial growth by the plant extract combinations [17–19].

The minimum inhibitory concentration of the individual and combined plant extracts was the lowest concentration that did not show any significant change in optical density values compared to the sterility control wells. The fractional inhibitory concentration values were calculated to determine the interaction between the two plant extracts.

The fractional inhibitory concentration index was calculated using the following formula:


$$\begin{array}{c} \text{FIC Index }(\Sigma \text{FIC})=\text{FICA}+\text{FICB} \end{array}$$

where

FICA = MIC of extract A in combination divided by MIC of extract A alone

FICB = MIC of extract B in combination divided by MIC of extract B alone

The interaction between the extracts was interpreted based on the calculated ΣFIC values. A ΣFIC value less than or equal to 0.5 indicated synergistic interaction. Values between 0.5 and 1.0 indicated additive interaction. Values between 1.0 and 4.0 indicated indifferent interaction. Values greater than 4.0 indicated antagonistic interaction.

### Biofilm formation and early biofilm inhibition assay method

Biofilm formation and inhibition were determined using the 96-well microtiter plate crystal violet biofilm quantitation assay as described in the Microtiter Dish Biofilm Formation Assay and further detailed in Quantitative and Qualitative Assessment Methods for Biofilm. This method was used to assess inhibition of biofilm formation and not biofilm destruction.

Overnight cultures of bacteria were prepared in sterile broth and standardized to approximately 1 x 10^6 CFU/mL. An inoculum of 100 microliters of standardized bacterial culture was placed in each well of a sterile flat-bottomed 96-well microtiter plate. The plate was incubated at 37 degrees Celsius for 4 hours to allow bacterial cells to adhere to the surface of the plate. This is the initial attachment phase of biofilm formation and is often used to allow bacterial cells to adhere stably to the surface of the plate prior to biofilm formation.

Following this, 100 µL of plant extract solutions, prepared in sterile broth at 25, 50, 75, and 100 mg/mL, were added to the wells. This range of concentrations was determined based on the range of the antimicrobial activity obtained during the determination of the minimum inhibitory concentration.

Control wells were included in the assay. Growth control wells had bacterial suspensions and sterile broth without plant extract, while media control wells had sterile broth without bacterial inoculum to ascertain the sterility of the medium. On the other hand, extract control wells had plant extract in sterile broth but without bacterial inoculum to ascertain that the plant extract does not cause background staining.

After addition of the extracts, the plates were incubated at 37 degrees Celsius for 24 hours to facilitate the formation of biofilms in the presence or absence of the extracts. After 24 hours of incubation, the liquid content of each well was carefully removed. The wells were gently washed with saline solution to remove any non-adherent bacterial cells. The plates were inverted and air-dried at room temperature for 10 to 15 minutes.

The biofilms were stained with 0.1 percent weight/volume crystal violet solution. Biofilms were stained at room temperature for 20 minutes. The unattached dye was removed by rinsing each well with deionized water to remove excess stain. The plates were air-dried at room temperature. To measure the optical density of the retained dye, 200 µL of 96 percent ethanol solution was added to each well to solubilize the retained crystal violet. The optical density of the retained dye was determined at 590 nm using a microplate reader. The optical density is an indication of the total biofilm biomass that is attached to each well surface [20,21].

Biofilm inhibition was calculated using the formula:


$$\begin{array}{c} \text{Biofilm Inhibition }(\%)=\left[1-\frac{𝐎𝐃𝐬𝐚𝐦𝐩𝐥𝐞}{\text{ODgrowth control}}\right]\times \text{100} \end{array}$$

Positive inhibition values show that there is a decrease in biofilm biomass compared to the control. Negative inhibition values show an increase in biofilm biomass compared to the control. This can happen as a response of bacteria to sub-inhibitory concentrations of antimicrobial compounds. The bacteria can increase their biofilm production as a response to this.

### Statistical analysis

All data were analyzed using GraphPad Prism 8.0.1 (GraphPad Software, USA) and Microsoft Excel. Results are expressed as the mean ± standard deviation (SD) of three independent replicates. The effects of extract concentration and bacterial strain on zone of inhibition and biofilm inhibition percentages were assessed with a two-way analysis of variance (ANOVA). Post hoc comparisons were made using Tukey’s Honestly Significant Difference (HSD) test. Statistical significance was determined at α ≤ 0.05.

## Results

### Phytochemical compounds

The qualitative phytochemical screening of the ethanolic extract of *Vernonia amygdalina* and *Senna siamea* detected the presence of alkaloids, saponins, tannins, flavonoids, glycosides, coumarins, triterpenoids, and phytosterols (Table 1). All compounds were detected in both plants, except glycosides which were found exclusively in *Vernonia amygdalina*.

**Table 1:** Qualitative and quantitative phytochemical composition of *Vernonia amygdalina* and *Senna siamea* leaf extracts (mg/g of dried extract). Values represent mean ± SD (n = 3). ND indicates not detected.

S/N	Phytochemical	*Vernonia amygdalina* (mg/g extract)	*Senna siamea* (mg/g extract)
1	Alkaloids	5.10 ± 0.25	4.50 ± 0.22
2	Flavonoids	12.50 ± 1.20	10.80 ± 1.10
3	Saponins	3.20 ± 0.35	2.90 ± 0.30
4	Glycosides	4.00 ± 0.20	ND
5	Coumarins	2.80 ± 0.15	2.40 ± 0.12
6	Triterpenoids	1.90 ± 0.05	1.70 ± 0.04
7	Phytosteroids	3.50 ± 0.18	3.00 ± 0.15
8	Tannins	1.50 ± 0.05	1.20 ± 0.03

Key

ND = Not detected.

Quantitative analysis showed that *Vernonia amygdalina* had higher concentrations of most phytochemicals compared to *Senna siamea*. Flavonoids were the most abundant in both extracts, with 12.50 ± 1.20 mg/g of dried extract in *Vernonia amygdalina* and 10.80 ± 1.10 mg/g of dried extract in *Senna siamea*.

### Antimicrobial susceptibility testing (AST)

#### Vernonia amygdalina.

*Vernonia amygdalina* ethanolic extract showed antibacterial activity against all pathogens, with the highest mean inhibition zone diameter (IZDs) of the ethanolic extract at 200 mg/mL against *E. coli* (IZD = 21.33 ± 4.45 mm), while non-ESBL-producing *Klebsiella pneumoniae* had the lowest susceptibility (IZD = 14.23 ± 3.39 mm) as shown in Table 2. A two-way ANOVA also showed a significant effect of concentration, F(3, 56) = 587.90, p < 0.0001; organism, F(6, 56) = 33.02, p < 0.0001; and a significant interaction between concentration and organism, F(18, 56) = 15.67, p < 0.0001 (S4–S6 Tables).

**Table 2:** Antimicrobial activity of *Vernonia amygdalina* ethanolic extract against selected pathogenic organisms measured as zones of inhibition (mm).

Test organism	200 mg/mL	100 mg/mL	50 mg/mL	25 mg/mL	Cipro 50 μg/mL	DMSO
*Salmonella typhi*	16.90 ± 0.27	10.73 ± 4.65	6.90 ± 1.84	1.90 ± 0.56	27	0
*Staphylococcus aureus*	18.97 ± 4.31	11.73 ± 3.48	4.97 ± 2.04	0.93 ± 0.92	29	0
*Escherichia coli*	21.33 ± 4.45	10.23 ± 4.62	5.00 ± 1.21	2.87 ± 1.56	27	0
*Escherichia coli* (ESBL)	19.40 ± 4.25	12.73 ± 3.51	5.60 ± 2.23	2.90 ± 1.38	26	0
*Klebsiella pneumoniae*	14.23 ± 3.39	11.60 ± 2.04	3.93 ± 2.17	1.00 ± 0.53	24	0
*Klebsiella pneumoniae* (ESBL) *Escherichia coli* ATCC 25922	16.07 ± 3.02 22.52 ± 2.11	11.36 ± 2.84 13.21 ± 3.62	5.40 ± 1.81 6.38 ± 1.99	3.00 ± 1.67 2.66 ± 1.03	29 31	0 0

Values are mean ± SD (n = 3). Cipro = Ciprofloxacin 50 μg/mL. DMSO = negative control.

#### Senna siamea.

The extract from *Senna siamea* showed clear dose-dependent antibacterial properties (Table 3). The maximum zones of inhibition observed were 200 mg/mL against *Klebsiella pneumoniae* (27.83 ± 3.87 mm) and *Salmonella typhi* (25.00 ± 1.51 mm). *Klebsiella pneumoniae* (ESBL) also recorded the lowest zone of inhibition (15.97 ± 2.32) at the highest concentration. A two-way ANOVA showed a significant effect of concentration, F(3, 56) = 733.60, p < 0.0001; organism, F(6, 56) = 32.22, p < 0.0001; and a significant interaction between concentration and organism, F(18, 56) = 12.84, p < 0.0001 (S1–S3 Tables).

**Table 3:** Antimicrobial activity of *Senna siamea* ethanolic extract against selected pathogenic organisms measured as zones of inhibition (mm).

Test Organism	200 mg/ mL	100 mg/mL	50 mg/mL	25 mg/mL	Cipro 50 μg/mL	DMSO
*Salmonella typhi*	25.00 ± 1.51	14.00 ± 1.30	5.17 ± 2.53	2.27 ± 2.14	27	0
*Staphylococcus aureus*	22.97 ± 1.16	15.00 ± 1.85	7.87 ± 2.58	3.80 ± 2.52	29	0
*Escherichia coli*	21.00 ± 2.62	10.00 ± 2.26	5.83 ± 2.32	3.03 ± 0.29	27	0
*Escherichia coli* (ESBL)	19.17 ± 3.61	13.07 ± 1.89	4.10 ± 1.50	5.97 ± 1.12	26	0
*Klebsiella pneumoniae*	27.83 ± 3.87	13.83 ± 2.76	8.80 ± 1.45	1.10 ± 0.56	24	0
*Klebsiella pneumoniae* (ESBL) *Escherichia coli* ATCC 25922	15.97 ± 2.32 24.21 ± 2.86	10.97 ± 3.58 14.83 ± 2.66	5.40 ± 1.81 7.92 ± 1.07	3.13 ± 1.43 3.39 ± 0.88	29 34	0 0

Values are mean ± SD (n = 3). Cipro = Ciprofloxacin 50 μg/mL. DMSO = negative control.

### Minimum inhibitory concentration (MIC) and minimum bactericidal concentration (MBC) of *Vernonia amygdalina* ethanolic extract

*Vernonia amygdalina* showed substantial antibacterial activity against the test organisms (Table 4). *Escherichia coli* recorded the highest MIC and MBC (41.67 ± 14.43, 100.00 ± 0.00) mg/mL, while *Klebsiella pneumoniae* (ESBL) recorded the lowest (MIC = 12.50 ± 0.00, MBC = 25.00 ± 0.00) mg/mL. All values are presented as the mean of three independent determinations.

**Table 4:** Minimum inhibitory concentration (MIC) and minimum bactericidal concentration (MBC) of *Vernonia amygdalina* ethanolic extract (mg/mL). Values represent the most consistent result across three replicates (n = 3).

Test organism	MIC (mg/mL)	MBC (mg/mL)
*Escherichia coli* ATCC 25922	12.5	25
*Salmonella typhi*	25	50
*Staphylococcus aureus*	25	50
*Escherichia coli*	50	100
*E. coli* (ESBL)	12.5	25
*Klebsiella pneumoniae*	12.5	25
*K. pneumoniae* (ESBL)	12.5	25

### Minimum inhibitory concentration (MIC) and minimum bactericidal concentration (MBC) of *Senna siamea* ethanolic extract

*Senna siamea* demonstrated the highest activity against *Klebsiella pneumoniae* (MIC = 6.25 mg/mL and MBC = 12.50 mg/mL) (Table 5), while non-ESBL-producing *E. coli* had the least susceptibility, needing higher concentrations to inhibit and kill bacterial growth (MIC = 25.00 mg/mL; MBC = 50.00 mg/mL). Values represent the most consistent result across three independent replicates.

**Table 5:** Minimum inhibitory concentration (MIC) and minimum bactericidal concentration (MBC) of *Senna siamea* ethanolic extract (mg/mL). Values represent the most consistent result across three replicates (n = 3).

Test organism	MIC (mg/mL)	MBC (mg/mL)
*Escherichia coli* ATCC 25922	6.25	12.5
*Salmonella typhi*	12.5	25
*Staphylococcus aureus*	12.5	25
*Escherichia coli*	25	50
*E. coli* (ESBL)	12.5	25
*Klebsiella pneumoniae*	6.25	12.5
*K. pneumoniae* (ESBL)	12.5	25

### Synergistic effects of *Senna siamea* and *Vernonia amygdalina* ethanolic extracts against test organisms

Table 6 shows the combined effect of the extract against the test organisms. Synergistic effects were observed against *E. coli*, while additive effects occurred with *S. typhi, S. aureus, and K. pneumoniae* (ESBL). Indifferent interactions were observed for ESBL-producing *E. coli* and non-ESBL *K. pneumoniae*.

**Table 6:** Fractional inhibitory concentration (FIC) indices for the combined extracts of *Senna siamea* and *Vernonia amygdalina.*

Test organism	FIC (*S. siamea*)	FIC (*V. amygdalina*)	ΣFIC	Interaction
*Salmonella typhi*	0.50	0.25	0.75	Additive
*Staphylococcus aureus*	0.50	0.25	0.75	Additive
*Escherichia coli*	0.25	0.125	0.375	Synergistic
*Escherichia coli* (ESBL)	1.00	1.00	2.00	Indifferent
*Klebsiella pneumoniae*	1.00	0.50	1.50	Indifferent
*K. pneumoniae* (ESBL)	0.50	0.50	1.00	Additive

Values represent FIC and the summed FIC for each organism.

### Antibiofilm activity of *Vernonia amygdalina* extract

The results on the antibiofilm activity of the *V. amygdalina* Extract showed a moderate inhibitory effect, with inhibition increasing with higher concentration (Table 7). Interestingly, at 25 mg/ml, the extract promoted the formation of biofilms (−4.32%) against *Staphylococcus aureus,* and at 100 mg/ml, the extract showed the highest biofilm inhibition (99.01%) against ESBL*-*producing *Klebsiella pneumoniae*. A two-way ANOVA also showed a significant effect of concentration, F(4, 60) = 259.20, p < 0.0001; organism, F(5, 60) = 27.53, p < 0.0001; and a significant interaction between concentration and organism, F(20, 60) = 9.23, p < 0.0001 (S10–S12 Tables).

**Table 7:** Antibiofilm activity (%) of *Vernonia amygdalina* ethanolic extract against selected pathogenic bacteria. Values are expressed as Mean ± SD (n = 3). Negative values indicate biofilm promotion.

Concentration (mg/mL)	*S. typhi*	*S. aureus*	*E. coli*	*E. coli* (ESBL)	*K. pneumoniae*	*K. pneumoniae* (ESBL)
25	44.08 ± 9.14	−4.32 ± 14.78	39.31 ± 12.63	41.81 ± 16.38	30.09 ± 19.52	35.58 ± 17.88
50	67.87 ± 8.64	50.61 ± 18.21	63.62 ± 14.76	62.33 ± 14.02	60.78 ± 15.87	58.00 ± 18.22
75	75.95 ± 10.23	90.52 ± 5.38	93.00 ± 4.12	86.57 ± 7.35	80.40 ± 10.14	74.84 ± 11.33
100	91.03 ± 4.28	94.16 ± 3.21	93.53 ± 3.67	95.65 ± 2.94	91.70 ± 5.06	99.01 ± 0.89

Experiments were performed in triplicate (n = 3).

### Antibiofilm activity of the *Senna siamea* ethanolic extract

*Senna siamea* extract showed a significant dose-dependent inhibition of biofilm formation (Table 8) on all test organisms. Inhibition was minimal at 25 mg/mL for *K. pneumoniae* and the ESBL strain (6.65% and 6.49%, respectively). Inhibition also progressively increased with concentration, with maximum levels reaching 99.72% (*Staphylococcus aureus* at 100 mg/mL) and 99.13% (*Salmonella typhi* at 100 mg/mL). A two-way ANOVA showed a significant effect of concentration, F(4, 60) = 200.60, p < 0.0001; organism, F(5, 60) = 18.50, p < 0.0001; and a significant interaction between concentration and organism, F(20, 60) = 8.93, p < 0.0001 (S7–S9 Tables).

**Table 8:** Antibiofilm activity (%) of *Senna siamea* ethanolic extract against selected pathogenic bacteria. Values are expressed as Mean ± SD (n = 3).

Concentration (mg/mL)	*S. typhi*	*S. aureus*	*E. coli*	*E. coli* (ESBL)	*K. pneumoniae*	*K. pneumoniae* (ESBL)
25	44.36 ± 10.28	34.26 ± 13.67	47.49 ± 13.21	25.26 ± 18.44	6.65 ± 19.11	6.49 ± 17.22
50	75.88 ± 9.66	46.11 ± 15.92	63.05 ± 14.37	59.92 ± 15.11	71.23 ± 13.84	34.38 ± 17.60
75	88.31 ± 6.72	68.43 ± 8.41	82.70 ± 5.93	80.29 ± 7.66	79.77 ± 9.08	53.61 ± 14.20
100	99.13 ± 2.18	99.72 ± 0.88	95.74 ± 2.96	91.75 ± 4.85	92.41 ± 5.37	61.78 ± 12.66

Experiments were performed in triplicate (n = 3).

## Discussion

The study provided the first comparative analysis of *V. amygdalina* and *S. siamea* against ESBL and non ESBLS producing bacteria in Ghana, including extract–extract potentiation and anti-biofilm profiling.

Medicinal plants contain chemical compounds that account for definite physiological and biochemical actions in the human body, and they are known as phytochemicals or phytocomponents [22]. The phytochemical analysis of *V. amygdalina* in the current study has revealed the presence of tannins, saponins, glycosides, flavonoids, triterpenoids, phytosteroids, coumarins, and alkaloids. These findings align with [22], who also found these compounds in the methanolic extract of the plant. However, other studies recorded some variations [23–25]. In contrast, in the *Senna siamea* extract, all the compounds screened were reported except for glycosides, which were only found in *Vernonia amygdalina*. These findings also confirm the results of [26], who reported all of these in their methanolic extracts of *Senna siamea*. Several studies also reported some variations [27–29].

Secondary metabolites are noted for the pharmacological and antimicrobial properties of medicinal plants [30]. Alkaloids have antibacterial properties by inhibiting bacterial cell division and regulating metabolism in bacteria, both innate plant defenses [31]. The alkaloids found in V. amygdalina have significant antimicrobial and anti-inflammatory properties [32]. Flavonoids offer antioxidant, antibacterial, anti-inflammatory, and positive cardiovascular effects [33], while saponins and tannins also enhance the medicinal properties of these plant extracts [34–36].

The antibacterial activity of the *V. amygdalina* ethanolic extract in this study indicates the possibility of it being a complementary treatment for bacterial infections, particularly against *Salmonella typhi*, *Staphylococcus aureus*, *Escherichia coli* and its ESBL, *Klebsiella pneumoniae*, and its ESBL. The highest zone of inhibition for *V. amygdalina* was recorded against *E. coli* (21.33 ± 4.45 mm). The antibacterial activity of the *V. amygdalina* ethanolic extract in this study confirms findings from previous studies on the antimicrobial susceptibility testing on the plant extract [22,23,37]. Also, the antibacterial activity of the *Senna siamea* ethanolic extract in this study confirms findings from previous studies on the antimicrobial susceptibility testing on the plant extract [38,39]. The highest zone of inhibition for *Senna siamea* was recorded against *K. pneumoniae* (27.83 ± 3.87 mm), indicating stronger efficacy. The results of this study emphasize the significance of investigating the therapeutic potential of medicinal plants due to increasing antibiotic resistance. These plants could offer a sustainable and cost-effective way of combating bacterial infections.

The determination of Minimum Inhibitory Concentration (MIC) and Minimum Bactericidal Concentration (MBC) provides further evidence of the effectiveness of the extracts, and both parameters serve as fundamental tools for evaluating the in vitro Antimicrobial Potential of antimicrobial agents [40]. The extract of *V. amygdalina* exhibited potent antibacterial effects against all of the organisms examined, while *S. siamea* demonstrated the greatest efficacy against *K. pneumoniae* (MIC = 6.25 mg/mL), which corresponds with the findings by [41].

The combination of *S. siamea* and *V. amygdalina* improved the antibacterial effect in all organisms evaluated. These results were also consistent with prior reports that documented a synergistic effect from the use of two or more combined extracts of plants [42–44].

Both extracts also exhibited concentration-dependent anti-biofilm (biofilm inhibition) activity in this study; *S. siamea* (100 mg/mL) produced a maximum of inhibition (99.72%) against *Staphylococcus aureus*, whereas *V. amygdalina* had the maximum inhibition (99.01%) against ESBL-producing *K. pneumoniae*. These findings support prior research documenting the biofilm inhibitory activity of medicinal plants [45–49]. It is interesting that at a sub-inhibitory concentration of 25 mg/mL, *V. amygdalina* also exerted a biofilm-enhancing effect against *S. aureus* (4.32% decrease in biofilm formation), demonstrating that plant constituents at sub-inhibitory levels can also act as mild stressors, stimulating biofilm production as part of the organism’s defense. Supporting this observation is the current literature regarding the use of plant polysaccharides (arabinogalactan, pectin, xylan), which have been found to stimulate biofilm production in *Bacillus subtilis* [45].

The studies have shown that the plant extracts have potential therapeutic properties with respect to the biofilms produced by multidrug-resistant bacteria and thus support their use as potential replacements for conventional therapies, which can help decrease the use of synthetic antibiotics and subsequent rates of resistance development. The results from the current study suggest that plant-derived products have potential as safer and more economical alternatives to antimicrobials; however, a limitation of this study is that the entire investigation was performed in vitro and may not directly correlate with in vivo systems. The absence of toxicology profiling or pharmacokinetics information prevents clinical applicability and needs to be addressed with further research.

### Conclusion

The study shows that both plant extracts have bioactive compounds that contribute to their antimicrobial activity. Both extracts exhibited antibacterial activity, with *Senna siamea* being more effective. The combination of the two plant extracts exhibited a synergistic effect against E. coli. The anti-biofilm effect of these plant extracts demonstrates the effectiveness of the extract, confirming bioactive compounds are involved in inhibiting biofilm formation. These extracts can be used to combat bacterial infections. Future studies, however, should establish efficacy, safety, and the optimal dose of the extracts of these plants.

## Supporting information

S1 Table Zone of inhibition – *Senna siamea* extract. (DOCX)

S2 Table Tukey post hoc test (Concentration). (DOCX)

S3 Table Tukey post hoc test (Organisms). (DOCX)

S4 Table Two-way ANOVA summary (Zone of inhibition – *Vernonia amygdalina* extract). (DOCX)

S5 Table Tukey post hoc test (Concentration). (DOCX)

S6 Table Tukey post hoc test (Organisms). (DOCX)

S7 Table Two-way ANOVA summary (Biofilm inhibition (OD) – *Senna siamea*). (DOCX)

S8 Table Tukey post hoc test (Concentration). (DOCX)

S9 Table Tukey post hoc test (Organisms). (DOCX)

S10 Table Two-way ANOVA summary (Biofilm inhibition (OD) – *Vernonia amygdalina).* (DOCX)

S11 Table Tukey post hoc test (Concentration). (DOCX)

S12 Table Tukey post hoc test (Organisms). (DOCX)

S1 Data Raw data. (ZIP)
