# Supplementary material for: Antimicrobial and anti-biofilm activities of Vernonia amygdalina and Senna siamea leaf extracts against pathogenic bacteria
Source: PLoS One. 2026 Oct 1;21(10):e0358840. doi: 10.1371/journal.pone.0358840 (PMC13630245; doi:10.1371/journal.pone.0358840)
Supplement: Supplementary file 1 [file pone.0358840.s014.pdf]

Comments from Reviewer 1

1. Authors should justify the novelty of their work. The antimicrobial and antibiofilm activities of the plants have been reported.
https://journaljocamr.com/index.php/JOCAMR/article/view/242
https://doi.org/10.58578/ajmsphr.v2i3.7038
https://doi.org/10.1101/2025.05.22.655479

2. Page 3 line 61; authors should clarify the statement 'Biofilms are organized bacterial communities that are secreted by the bacteria themselves in an extracellular matrix'.
3. Provide adequate information on the two plants
4. The justification for the use of the 2 plants is weak and therefore, author should give a strong justification for the use of the plants
5. These plants have been reported to have antimicrobial activity, but no statement is on that in the introduction
6. The introduction suggests focussing on ESBL organisms, however there are other non-ESBL producing bacteria were employed in the study as well, authors should provide clarity
7. Are the organisms clinical or typed strains?
8. Authors should indicate the cork borer size used
9. Authors should explain why only one Gram-positive organism was used
10. Authors should indicate the extractive yield
11. How was the extract sterilized?
12. At what temperature was rotary evaporator set?
13. Page 5, line 100 -102; the information provided is not clear. Authors should revise to bring clarity
14. A summary table should be provided to indicate the specific tests performed for both the qualitative and quantitative identification of the stated phytochemicals
15. Line 113 how was the air-drying done
16. Line 115 change, was used to treat each well to was transferred into the appropriate well
17. Line 124 authors should state the concentrations of the extract used, volume of nutrient broth, and authors should indicate controls
18. Why did authors allow the organism to attach for 4 h? biofilm formation assay should be independent and not attached to inhibition assay
19. Line 169 were the wells allowed to dry before the CV was added?
20. Authors should indicate whether the solubilised CV were transferred into a

separate 96 well plate before reading
21. Line 174: authors should clarify if it is OD of treated or OD of sample
22. Table 1: authors should check the key
23. Table 1: Values represent mean ± SD (n = 3). ND indicates not detected, should be added to key below the table
24. Tables 2 and 3: no standard deviation values for cipro
25. Tables 4 and 5: If MIC was done in 2-fold serial dilution how come authors are getting those values?
26. Line 256: authors should clarify how the extract promoted the formation of biofilm
27. Table 7: Values represent the percentage inhibition of biofilm formation at different concentrations (mg/mL). Negative values indicate biofilm promotion; authors should bring it below the table. They should do same for all
28. Line 288, authors should add the name of the author. E.g. Sampson et al. [22].
29. Biofilm inhibition assays use sub-MICs of extract. The authors should clarify why the used concentrations range from 25 to 100 mg/mL which is within the MIC range of the extracts. Hence, it is not possible to conclude that the activity may be due to biofilm inhibition.

Comments from Reviewer 2

The paper is a simple and straight forward paper, which is not too novel but very important in the era of antimicrobial resistance. The authors could have looked at more plant extracts than just the two they looked at in the discussion some of the statements are not complete. for instance on page 19, line 288, the authors wrote These findings align with [22],.... instead of writing 'these findings align with Ali et al [22]. There are quite a number of them in the discussion which need to be corrected.

Comments from Reviewer 3

Generally clear writing with logical organization. Appropriate use of tables for data presentation. Discussion adequately contextualizes findings within existing literature. Please address the following.

- Line 346-347- The combination of plant extracts with ciprofloxacin exhibited a greater synergistic effect as well. you did not test any extract-ciprofloxacin

combinations. The synergy testing (Lines 132-157, Table 6) (Lines 116, 209, 221). The actual finding was synergy between the two extracts against E.coli only, Table 6, Line 251.

- Lines 30-31- Senna siamea, there was a significant difference only among the organisms, $p = 0.9161$ (appears only in abstract). The Results section (Lines 216-218). The discrepancy between $p = 0.9161$ (abstract) and $p = 0.231$ (results) must also be explained.

- Not a statistician, but ANOVA results for the two extracts show incompatible degrees of freedom structures. The denominator df differ a lot (48 vs. 15). Explain. Also, no details on interaction data? Include all the stats as supplementary files.

- Add dose-response figures. At least one figure in the whole MS.

- Abstract (Lines 27-28): S. siamea MIC range reported as 8.33 ± 3.61-33.33 ± 14.43 mg/mL, Table 5 (Line 241): Shows MIC for E. coli ATCC 25922 as 6.25 ± 0.00 mg/mL

- Line 100 - sterilized extract- how was this done?

- Report extraction yield, describe the sterilization procedure, and detail how working concentrations were prepared from the dried extract.

- Lines 84-89- can you add more details

- Check the GPS coordinates appear reversed (Line 86-87)

- Lines 158-176- This experimental design tests biofilm development inhibition and not biofilm formation prevention. The terminology throughout the manuscript should be streamlined. It mixes these distinct mechanisms. Pre-formed (mature) biofilm disruption/eradication was not tested.

- Incomplete MIC/MBC Methodology (Lines 121-131)- full details, aerobic, inoclum density etc.

- Table 1, Lines 193-197- The quantitative values are reported in mg/g, mg per gram of dried extract or mg per gram of original plant material. Need to be revised in the MS.

- Why no positive controls to validate the assay system.

- Discussion more of Biofilm Promotion (Lines 257, 327-331). Sub-inhibitory concentrations promoting biofilm formation could have adverse therapeutic implications.

- Soften the language (e.g., "To our knowledge, this is among the first...").

- include Strain Information for Clinical Isolates- Lines 78-79

- Lines 204-206, 216-218, provide ANOVA tables as supplementary material
- Post-hoc test results not shown, Line 183 mentions LSD, Report pairwise comparisons for significant effects, as supplementary material

- Specify whether n=3 represents independent experiments (biological) or within-experiment replicates (technical)

- Ciprofloxacin values lack SD (reported as single values)

- Tables 7 & 8: Standard deviations not provided for biofilm inhibition percentages despite triplicate experiments

- Reference 31: What? business branding text (The future of purpose-driven branding) ChatGPT?

- Reference 9 and 28
