# Supplementary material for: Antimicrobial and anti-biofilm activities of Vernonia amygdalina and Senna siamea leaf extracts against pathogenic bacteria
Source: PLoS One. 2026 Oct 1;21(10):e0358840. doi: 10.1371/journal.pone.0358840 (PMC13630245; doi:10.1371/journal.pone.0358840)
Supplement: Supplementary file 2 [file pone.0358840.s001.docx]

1. Zone of Inhibition – Senna siamea Extract

**Table S1**

Two-Way ANOVA Summary

Source of Variation	% Total Variation	SS	df	MS	F (dfn, dfd)	p-value	Sig.

Interaction	8.23	1247.30	18	69.29	F(18, 56) = 12.84	<0.0001	****

Concentration	78.45	11876.20	3	3958.70	F(3, 56) = 733.60	<0.0001	****

Organism	6.89	1043.20	6	173.90	F(6, 56) = 32.22	<0.0001	****

Residual	6.43	302.10	56	5.40	—	—	—
