# Supplementary material for: Antimicrobial and anti-biofilm activities of Vernonia amygdalina and Senna siamea leaf extracts against pathogenic bacteria
Source: PLoS One. 2026 Oct 1;21(10):e0358840. doi: 10.1371/journal.pone.0358840 (PMC13630245; doi:10.1371/journal.pone.0358840)
Supplement: Supplementary file 3 [file pone.0358840.s002.docx]

**Table S2**

Tukey Post Hoc Test (Concentration)

Comparison	Mean Diff.	95% CI	p-value	Sig.

200 vs 100	8.46	6.89 to 10.02	<0.0001	****

200 vs 50	13.23	11.67 to 14.80	<0.0001	****

200 vs 25	16.79	15.23 to 18.35	<0.0001	****

100 vs 50	4.78	3.21 to 6.34	<0.0001	****

100 vs 25	8.33	6.77 to 9.90	<0.0001	****

50 vs 25	3.56	1.99 to 5.12	0.0002	***
