# Supplementary material for: Antimicrobial and anti-biofilm activities of Vernonia amygdalina and Senna siamea leaf extracts against pathogenic bacteria
Source: PLoS One. 2026 Oct 1;21(10):e0358840. doi: 10.1371/journal.pone.0358840 (PMC13630245; doi:10.1371/journal.pone.0358840)
Supplement: Supplementary file 4 [file pone.0358840.s003.docx]

**Table S3**

Tukey Post Hoc Test (Organisms)

Comparison	Mean Diff.	95% CI	p-value	Sig.

S. typhi vs S. aureus	1.23	-0.46 to 2.92	0.3456	ns

S. typhi vs E. coli	2.57	0.88 to 4.26	0.0023	**

S. typhi vs E. coli ESBL	3.12	1.43 to 4.81	0.0004	***

S. typhi vs K. pneumoniae	-0.46	-2.15 to 1.23	0.9234	ns

S. typhi vs K. pneumoniae ESBL	4.23	2.54 to 5.92	<0.0001	****

S. typhi vs E. coli ATCC	0.79	-0.90 to 2.48	0.7123	ns

S. aureus vs E. coli	1.33	-0.36 to 3.02	0.2345	ns

S. aureus vs E. coli ESBL	1.89	0.20 to 3.58	0.0234	*

S. aureus vs K. pneumoniae	-1.69	-3.38 to 0.00	0.0501	ns

S. aureus vs K. pneumoniae ESBL	3.00	1.31 to 4.69	0.0006	***

S. aureus vs E. coli ATCC	-0.45	-2.14 to 1.25	0.9456	ns
