# Supplementary material for: Antimicrobial and anti-biofilm activities of Vernonia amygdalina and Senna siamea leaf extracts against pathogenic bacteria
Source: PLoS One. 2026 Oct 1;21(10):e0358840. doi: 10.1371/journal.pone.0358840 (PMC13630245; doi:10.1371/journal.pone.0358840)
Supplement: Supplementary file 5 [file pone.0358840.s004.docx]

**Table S4**

Two-Way ANOVA Summary

Source of Variation	% Total Variation	SS	df	MS	F (dfn, dfd)	p-value	Sig.

Interaction	11.57	1456.80	18	80.93	F(18, 56) = 15.67	<0.0001	****

Concentration	72.34	9108.40	3	3036.10	F(3, 56) = 587.90	<0.0001	****

Organism	8.12	1023.10	6	170.50	F(6, 56) = 33.02	<0.0001	****

Residual	7.97	289.20	56	5.16	—	—	—
