# Supplementary material for: Antimicrobial and anti-biofilm activities of Vernonia amygdalina and Senna siamea leaf extracts against pathogenic bacteria
Source: PLoS One. 2026 Oct 1;21(10):e0358840. doi: 10.1371/journal.pone.0358840 (PMC13630245; doi:10.1371/journal.pone.0358840)
Supplement: Supplementary file 6 [file pone.0358840.s005.docx]

**Table S5**

Tukey Post Hoc Test (Concentration)

Comparison	Mean Diff.	95% CI	p-value	Sig.

200 vs 100	6.23	4.71 to 7.76	<0.0001	****

200 vs 50	10.57	9.05 to 12.09	<0.0001	****

200 vs 25	13.46	11.93 to 14.98	<0.0001	****

100 vs 50	4.33	2.81 to 5.86	<0.0001	****

100 vs 25	7.22	5.70 to 8.74	<0.0001	****

50 vs 25	2.89	1.37 to 4.41	0.0003	***
