# Supplementary material for: Antimicrobial and anti-biofilm activities of Vernonia amygdalina and Senna siamea leaf extracts against pathogenic bacteria
Source: PLoS One. 2026 Oct 1;21(10):e0358840. doi: 10.1371/journal.pone.0358840 (PMC13630245; doi:10.1371/journal.pone.0358840)
Supplement: Supplementary file 7 [file pone.0358840.s006.docx]

**Table S6**

Tukey Post Hoc Test (Organisms)

Comparison	Mean Diff.	95% CI	p-value	Sig.

S. typhi vs S. aureus	2.12	0.46 to 3.79	0.0089	**

S. typhi vs E. coli	1.46	-0.21 to 3.12	0.1234	ns

S. typhi vs E. coli ESBL	1.79	0.12 to 3.46	0.0345	*

S. typhi vs K. pneumoniae	3.23	1.57 to 4.90	0.0003	***

S. typhi vs K. pneumoniae ESBL	2.57	0.90 to 4.23	0.0023	**

S. typhi vs E. coli ATCC	0.23	-1.43 to 1.90	0.9876	ns

S. aureus vs E. coli	-0.67	-2.33 to 1.00	0.7890	ns

S. aureus vs E. coli ESBL	-0.33	-2.00 to 1.33	0.9567	ns

S. aureus vs K. pneumoniae	1.11	-0.56 to 2.78	0.3456	ns

S. aureus vs K. pneumoniae ESBL	0.44	-1.22 to 2.11	0.9234	ns

S. aureus vs E. coli ATCC	-1.89	-3.56 to -0.22	0.0234	*
