# Supplementary material for: Antimicrobial and anti-biofilm activities of Vernonia amygdalina and Senna siamea leaf extracts against pathogenic bacteria
Source: PLoS One. 2026 Oct 1;21(10):e0358840. doi: 10.1371/journal.pone.0358840 (PMC13630245; doi:10.1371/journal.pone.0358840)
Supplement: Supplementary file 8 [file pone.0358840.s007.docx]

**Table S7**

Two-Way ANOVA Summary

Source of Variation	% Total Variation	SS	df	MS	F (dfn, dfd)	p-value	Sig.

Interaction	15.23	0.3847	20	0.0192	F(20, 60) = 8.93	<0.0001	****

Concentration	68.45	1.7289	4	0.4322	F(4, 60) = 200.60	<0.0001	****

Organism	7.89	0.1993	5	0.0399	F(5, 60) = 18.50	<0.0001	****

Residual	8.42	0.1293	60	0.0022	—	—	—
