# Supplementary material for: Antimicrobial and anti-biofilm activities of Vernonia amygdalina and Senna siamea leaf extracts against pathogenic bacteria
Source: PLoS One. 2026 Oct 1;21(10):e0358840. doi: 10.1371/journal.pone.0358840 (PMC13630245; doi:10.1371/journal.pone.0358840)
Supplement: Supplementary file 9 [file pone.0358840.s008.docx]

**Table S8**

Tukey Post Hoc Test (Concentration)

Comparison	Mean Diff.	95% CI	p-value	Sig.

Control vs 25	0.3456	0.2789–0.4123	<0.0001	****

Control vs 50	0.5234	0.4567–0.5901	<0.0001	****

Control vs 75	0.6789	0.6122–0.7456	<0.0001	****

Control vs 100	0.8123	0.7456–0.8790	<0.0001	****

25 vs 50	0.1778	0.1111–0.2445	<0.0001	****

25 vs 75	0.3333	0.2666–0.4000	<0.0001	****

25 vs 100	0.4667	0.4000–0.5334	<0.0001	****

50 vs 75	0.1555	0.0888–0.2222	<0.0001	****

50 vs 100	0.2889	0.2222–0.3556	<0.0001	****

75 vs 100	0.1334	0.0667–0.2001	0.0001	***
