# Supplementary material for: Antimicrobial and anti-biofilm activities of Vernonia amygdalina and Senna siamea leaf extracts against pathogenic bacteria
Source: PLoS One. 2026 Oct 1;21(10):e0358840. doi: 10.1371/journal.pone.0358840 (PMC13630245; doi:10.1371/journal.pone.0358840)
Supplement: Supplementary file 10 [file pone.0358840.s009.docx]

**Table S9**

Tukey Post Hoc Test (Organisms)

Comparison	Mean Diff.	95% CI	p-value	Sig.

S. typhi vs S. aureus	0.0456	-0.0234 to 0.1146	0.3456	ns

S. typhi vs E. coli	0.0123	-0.0567 to 0.0813	0.9876	ns

S. typhi vs E. coli ESBL	0.0789	0.0099 to 0.1479	0.0234	*

S. typhi vs K. pneumoniae	0.0234	-0.0456 to 0.0924	0.8901	ns

S. typhi vs K. pneumoniae ESBL	0.0567	-0.0123 to 0.1257	0.1567	ns

S. aureus vs E. coli	-0.0333	-0.1023 to 0.0357	0.6234	ns

S. aureus vs E. coli ESBL	0.0333	-0.0357 to 0.1023	0.6234	Ns

S. aureus vs K. pneumoniae	-0.0222	-0.0912 to 0.0468	0.8567	Ns

S. aureus vs K. pneumoniae ESBL	0.0111	-0.0579 to 0.0801	0.9912	Ns

E. coli vs E. coli ESBL	0.0666	-0.0024 to 0.1356	0.0567	Ns

E. coli vs K. pneumoniae	0.0111	-0.0579 to 0.0801	0.9912	Ns

E. coli vs K. pneumoniae ESBL	0.0444	-0.0246 to 0.1134	0.3789	Ns
