# Supplementary material for: Antimicrobial and anti-biofilm activities of Vernonia amygdalina and Senna siamea leaf extracts against pathogenic bacteria
Source: PLoS One. 2026 Oct 1;21(10):e0358840. doi: 10.1371/journal.pone.0358840 (PMC13630245; doi:10.1371/journal.pone.0358840)
Supplement: Supplementary file 11 [file pone.0358840.s010.docx]

**Table S10**

Two-Way ANOVA Summary

Source of Variation	% Total Variation	SS	df	MS	F (dfn, dfd)	p-value	Sig.

Interaction	12.68	0.3124	20	0.0156	F(20, 60) = 9.23	<0.0001	****

Concentration	71.23	1.7534	4	0.4384	F(4, 60) = 259.20	<0.0001	****

Organism	9.46	0.2328	5	0.0466	F(5, 60) = 27.53	<0.0001	****

Residual	6.63	0.1015	60	0.0017	—	—	—
