# Supplementary material for: Antimicrobial and anti-biofilm activities of Vernonia amygdalina and Senna siamea leaf extracts against pathogenic bacteria
Source: PLoS One. 2026 Oct 1;21(10):e0358840. doi: 10.1371/journal.pone.0358840 (PMC13630245; doi:10.1371/journal.pone.0358840)
Supplement: Supplementary file 12 [file pone.0358840.s011.docx]

**Table S11**

Tukey Post Hoc Test (Concentration)

Comparison	Mean Diff.	95% CI	p-value	Sig.

Control vs 25	0.3789	0.3123–0.4455	<0.0001	****

Control vs 50	0.5567	0.4901–0.6233	<0.0001	****

Control vs 75	0.7012	0.6346–0.7678	<0.0001	****

Control vs 100	0.8456	0.7790–0.9122	<0.0001	****

25 vs 50	0.1778	0.1112–0.2444	<0.0001	****

25 vs 75	0.3223	0.2557–0.3889	<0.0001	****

25 vs 100	0.4667	0.4001–0.5333	<0.0001	****

50 vs 75	0.1445	0.0779–0.2111	<0.0001	****

50 vs 100	0.2889	0.2223–0.3555	<0.0001	****

75 vs 100	0.1444	0.0778–0.2110	<0.0001	****
