# Supplementary material for: Antimicrobial and anti-biofilm activities of Vernonia amygdalina and Senna siamea leaf extracts against pathogenic bacteria
Source: PLoS One. 2026 Oct 1;21(10):e0358840. doi: 10.1371/journal.pone.0358840 (PMC13630245; doi:10.1371/journal.pone.0358840)
Supplement: Supplementary file 13 [file pone.0358840.s012.docx]

**Table S12**

Tukey Post Hoc Test (Organisms)

Comparison	Mean Diff.	95% CI	p-value	Sig.

S. typhi vs S. aureus	0.0234	-0.0456 to 0.0924	0.8567	ns

S. typhi vs E. coli	-0.0123	-0.0813 to 0.0567	0.9876	ns

S. typhi vs E. coli ESBL	0.0345	-0.0345 to 0.1035	0.5678	ns

S. typhi vs K. pneumoniae	0.0567	-0.0123 to 0.1257	0.1567	ns

S. typhi vs K. pneumoniae ESBL	0.0456	-0.0234 to 0.1146	0.3456	ns

S. aureus vs E. coli	-0.0357	-0.1047 to 0.0333	0.5234	ns

S. aureus vs E. coli ESBL	0.0111	-0.0579 to 0.0801	0.9912	ns

S. aureus vs K. pneumoniae	0.0333	-0.0357 to 0.1023	0.6234	ns

S. aureus vs K. pneumoniae ESBL	0.0222	-0.0468 to 0.0912	0.8901	ns

E. coli vs E. coli ESBL	0.0468	-0.0222 to 0.1158	0.3012	ns

E. coli vs K. pneumoniae	0.0690	-0.0000 to 0.1380	0.0501	ns

E. coli vs K. pneumoniae ESBL	0.0579	-0.0111 to 0.1269	0.1345	ns
