# Supplementary material for: Antimicrobial and anti-biofilm activities of Vernonia amygdalina and Senna siamea leaf extracts against pathogenic bacteria
Source: PLoS One. 2026 Oct 1;21(10):e0358840. doi: 10.1371/journal.pone.0358840 (PMC13630245; doi:10.1371/journal.pone.0358840)
Supplement: Supplementary file 14 [file pone.0358840.s016.docx]

**Responses to Reviewer’s Comments**

| **Reviewer’s Comment / Issue** | **Author’s Response** | **Page number** |
| --- | --- | --- |
| Novelty of the study is not justified. | Addressed. The introduction was revised to clearly explain the relevance of the study. The novelty claim was moderated and supported with existing literature. The study aim was clarified to include both ESBL and non-ESBL organisms. | 4 |
| Weak justification for plant selection | Addressed. Justification for selecting *Vernonia amygdalina* and *Senna siamea* was added based on ethnomedicinal use and reported antimicrobial properties. | 4 |
| Prior antimicrobial studies were not cited. | Addressed. Relevant studies on antimicrobial and antibiofilm activity of both plants were incorporated. | 4 |
| The organism source is not clear. | Addressed. Sources of isolates and strain details were clearly stated, including reference strains. | 5 |
| Lack of reference strain information | Addressed. A reference strain such as *E. coli* ATCC 25922 was included. | 5 |
| Extraction procedure incomplete | Addressed. Extraction yield, drying method, rotary evaporator temperature, and sterilization method were added. | 6 & 7 |
| Preparation of concentrations unclear | Addressed. Stock and working solution preparation were clearly described. | 7 |
| The MIC method is not fully described. | Addressed. Full broth microdilution method, inoculum density, and incubation conditions were added. | 9 |
| Controls not described | Addressed. Positive and negative controls were clearly defined. | 11 |
| Biofilm assay unclear | Addressed. Assay procedure was clarified. Study defined as early biofilm inhibition. Washing, drying, and OD measurement were explained. | 12 & 13 |
| Biofilm promotion is not explained. | Addressed. Observed promotion at low concentrations was discussed scientifically in the results. | 21 |
| Synergy interpretation incorrect | Addressed. Incorrect claims were removed. Conclusions revised to reflect actual findings. | 20 |
| Statistical inconsistencies | Addressed. Data were reanalyzed. P-values corrected and made consistent across sections. | 14 |
| Missing ANOVA and post hoc tests | Addressed. ANOVA tables and post hoc results were added as supplementary material. | 34 |
| Tables lack SD and clarity. | Addressed. All tables updated to include mean ± SD. Legends and units clarified. | 15, 16, 18, 22 |
| Phytochemical units unclear | Addressed. Units were defined, and the table was revised accordingly. | 15 |
| No figures included | Addressed. Dose-dependent curves and relevant figures were added. | 34 |
| Raw data not provided | Addressed. Raw datasets and replicates were added as supplementary files. | 34 - 41 |
| Poor referencing and citation errors | Addressed. All references were verified, corrected, and formatted. Missing citations were added. | Across entire document |
| Writing and formatting issues | Addressed. The entire manuscript was edited for grammar, clarity, and consistency. | Across entire document |
