# Supplementary material for: Antimicrobial and anti-biofilm activities of Vernonia amygdalina and Senna siamea leaf extracts against pathogenic bacteria
Source: PLoS One. 2026 Oct 1;21(10):e0358840. doi: 10.1371/journal.pone.0358840 (PMC13630245; doi:10.1371/journal.pone.0358840)
Supplement: Supplementary file 15 [file pone.0358840.s017.docx]

**Response to Reviewers**

*Manuscript: Antimicrobial and Anti-Biofilm Activities of Vernonia amygdalina and Senna siamea Leaf Extracts against Pathogenic Bacteria*

We thank Reviewer #3 for the careful re-review and for identifying the remaining inconsistencies. We have addressed each point below and revised the manuscript accordingly. A point-by-point response follows, indicating the specific location of each change in the revised manuscript.

**Reviewer #2**

No response was provided by Reviewer #2; no corresponding changes were required.

**Reviewer #3**

| **Reviewer Comment** | **Author Response** | **Location of Change** |
| --- | --- | --- |
| Reviewer #3 (Point 1): The extract-plus-ciprofloxacin synergy was never tested; the only synergy demonstrated was between the two plant extracts against E. coli (Table 6). The Abstract was corrected previously, but the Conclusion still made the ciprofloxacin claim. | We thank the reviewer for catching this inconsistency. The Conclusion has been revised to remove the claim of extract-plus-ciprofloxacin synergy. It now states only that the combination of the two plant extracts exhibited a synergistic effect against E. coli, consistent with the FIC index data reported in Table 6. No claim regarding ciprofloxacin combination synergy remains anywhere in the manuscript. | Conclusion section, paragraph 1 |
| Reviewer #3 (Point 2): The MIC ANOVA reported in the Abstract (p = 0.9161) does not match the Results (p = 0.231), and no corresponding analysis appears anywhere else in the manuscript. Provide the analysis or delete it. | We have removed the MIC ANOVA statement from the Abstract. MIC values are ordinal, two-fold dilution endpoints (e.g., 6.25, 12.5, 25, 50 mg/mL) rather than continuous measurements, and several test organisms in our data show zero variance across replicates (SD = 0.00). These properties mean the assumptions underlying ANOVA (continuous, normally distributed data with estimable within-group error) are not met, and we do not have grounds to report a valid inferential statistic for the MIC data. Consistent with common practice in antimicrobial susceptibility reporting, MIC and MBC values are now presented descriptively (mean +/- SD, or the most consistent value across replicates) in the Abstract, Results, and Discussion, without an accompanying ANOVA or p-value. | Abstract |
| Reviewer #3 (Point 3): Full ANOVA tables are now in the Supplement, but the Methods state that a Least Significant Difference (LSD) post hoc test was used, while every table reports Tukey's test. | This inconsistency has been corrected. The Statistical Analysis subsection of the Methods now states that post hoc comparisons were made using Tukey's Honestly Significant Difference (HSD) test, which matches the post hoc test reported in every ANOVA table in the manuscript and Supplement. | Methods, Statistical Analysis subsection |
| Reviewer #3 (Point 4): The Abstract reports an MIC range of 8.33-33.33 mg/mL for Senna siamea, while Table 5 shows a value of 6.25 mg/mL. The text, Table 5, and Discussion give different MIC values; these need to be reconciled. | The MIC values for Senna siamea have been reconciled across the entire manuscript. The Abstract, Results narrative, and Discussion now all report the same range, 6.25 to 25.00 mg/mL, matching the values presented in Table 5 exactly. No discrepant MIC figures for Senna siamea remain in the manuscript. | Abstract; Results narrative preceding Table 5; Discussion |
| Reviewer #3 (Point 5): Please clarify whether the phytochemical concentrations (mg/g) are expressed per dried extract or per plant material. | This has been clarified. A statement has been added specifying that quantitative phytochemical values are expressed as mg per gram of the dried crude extract (not per unit mass of raw plant material). The units in the Table 1 caption and column headers, as well as in the accompanying narrative text, have been updated to explicitly state "mg/g of dried extract" throughout. | Results, Phytochemical Composition subsection; Table 1 caption and column headers |
| Reviewer #3 (Point 6): References 9 and 28 are incomplete/improperly formatted; the reviewer suggested using Zotero or Google Scholar to correct the citations. | Both references have been corrected using the original source records. Reference 9 (Singh, Lalbiaktluangi, Zomuansangi, Srivastava, Yadav, and Gupta, 2024) now includes the full author list, book chapter title, editors/volume title, publisher, page range, and DOI. Reference 28 (Hassan, Nasiru, Malut, Abdulkadir, and Ali, 2015) now includes the full author list, correct journal name (Journal of Pharmacognosy and Phytotherapy), volume, issue, page range, and DOI. | Reference list, items 9 and 28 |

All changes described above are also highlighted in yellow in the accompanying Manuscript_Corrected_Version_Highlighted.docx file for the reviewer's convenience.
