# Supplementary material for: Antimicrobial and anti-biofilm activities of Vernonia amygdalina and Senna siamea leaf extracts against pathogenic bacteria
Source: PLoS One. 2026 Oct 1;21(10):e0358840. doi: 10.1371/journal.pone.0358840 (PMC13630245; doi:10.1371/journal.pone.0358840)
Supplement: Supplementary file 16 [file pone.0358840.s013.zip › Raw data/THESIS RAW DATA.docx]

**Zone of Inhibition (mm) of *Vernonia amygdalina* Extract**

| Organism | Concentration | Rep 1 | Rep 2 | Rep 3 |
| --- | --- | --- | --- | --- |
| *Salmonella typhi* | 200 mg/mL | 16.60 | 17.12 | 16.98 |
|  | 100 mg/mL | 14.50 | 12.17 | 5.52 |
|  | 50 mg/mL | 4.84 | 7.47 | 8.39 |
|  | 25 mg/mL | 1.27 | 2.07 | 2.35 |
|  | Cipro 50 µg/mL | 27.41 | 27.16 | 26.44 |
|  | DMSO | 0.00 | 0.00 | 0.00 |
| *Staphylococcus aureus* | 200 mg/mL | 14.14 | 22.46 | 20.31 |
|  | 100 mg/mL | 7.83 | 14.55 | 12.81 |
|  | 50 mg/mL | 2.69 | 6.62 | 5.60 |
|  | 25 mg/mL | 1.85 | 0.93 | 0.01 |
|  | Cipro 50 µg/mL | 29.41 | 29.16 | 28.44 |
|  | DMSO | 0.00 | 0.00 | 0.00 |
| *Escherichia coli* | 200 mg/mL | 16.35 | 22.71 | 24.93 |
|  | 100 mg/mL | 5.06 | 11.66 | 13.97 |
|  | 50 mg/mL | 3.64 | 5.38 | 5.98 |
|  | 25 mg/mL | 1.12 | 3.35 | 4.13 |
|  | Cipro 50 µg/mL | 26.44 | 27.16 | 27.41 |
|  | DMSO | 0.00 | 0.00 | 0.00 |
| *E. coli* (ESBL) | 200 mg/mL | 14.64 | 22.84 | 20.72 |
|  | 100 mg/mL | 8.80 | 15.57 | 13.82 |
|  | 50 mg/mL | 3.10 | 7.41 |  |
|  | 25 mg/mL | 1.35 | 3.33 | 4.02 |
|  | Cipro 50 µg/mL | 25.44 | 26.16 | 26.41 |
|  | DMSO | 0.00 | 0.00 | 0.00 |
| *Klebsiella pneumoniae* | 200 mg/mL | 10.43 | 16.98 | 15.28 |
|  | 100 mg/mL | 13.25 | 12.23 | 9.32 |
|  | 50 mg/mL | 5.69 | 4.60 | 1.50 |
|  | 25 mg/mL | 0.41 | 1.16 | 1.43 |
|  | Cipro 50 µg/mL | 23.44 | 24.16 | 24.41 |
|  | DMSO | 0.00 | 0.00 | 0.00 |
| *K. pneumoniae* (ESBL) | 200 mg/mL | 12.69 | 18.52 | 17.01 |
|  | 100 mg/mL | 13.66 | 12.24 | 8.18 |
|  | 50 mg/mL | 3.37 | 6.87 | 5.96 |
|  | 25 mg/mL | 1.13 | 4.35 | 3.52 |
|  | Cipro 50 µg/mL | 28.44 | 29.16 | 29.41 |
|  | DMSO | 0.00 | 0.00 | 0.00 |
| *E. coli* ATCC 25922 | 200 mg/mL | 20.16 | 24.23 | 23.17 |
|  | 100 mg/mL | 16.14 | 14.33 | 9.16 |
|  | 50 mg/mL | 7.99 | 7.00 | 4.15 |
|  | 25 mg/mL | 1.51 | 3.49 | 2.98 |
|  | Cipro 50 µg/mL | 30.44 | 31.16 | 31.41 |
|  | DMSO | 0.00 | 0.00 | 0.00 |

**Zone of Inhibition (mm) of *Senna siamea* Extract**

| Organism | Concentration | Rep 1 | Rep 2 | Rep 3 |
| --- | --- | --- | --- | --- |
| *Salmonella typhi* | 200 mg/mL | 23.60 | 24.80 | 26.60 |
|  | 100 mg/mL | 12.79 | 15.38 | 13.83 |
|  | 50 mg/mL | 7.85 | 4.84 | 2.82 |
|  | 25 mg/mL | 4.54 | 1.99 | 0.28 |
|  | Cipro 50 µg/mL | 26.44 | 27.14 | 27.16 |
|  | DMSO | 0.00 | 0.00 | 0.00 |
| *Staphylococcus aureus* | 200 mg/mL | 21.89 | 24.20 | 22.82 |
|  | 100 mg/mL | 13.28 | 16.96 | 14.76 |
|  | 50 mg/mL | 5.47 | 7.53 | 10.60 |
|  | 25 mg/mL | 6.47 | 3.47 | 1.46 |
|  | Cipro 50 µg/mL | 28.44 | 29.16 | 29.41 |
|  | DMSO | 0.00 | 0.00 | 0.00 |
| *Escherichia coli* | 200 mg/mL | 18.56 | 23.78 | 20.66 |
|  | 100 mg/mL | 7.90 | 12.40 | 9.71 |
|  | 50 mg/mL | 3.67 | 8.29 | 5.53 |
|  | 25 mg/mL | 3.34 | 2.99 | 2.76 |
|  | Cipro 50 µg/mL | 26.44 | 27.16 | 27.41 |
|  | DMSO | 0.00 | 0.00 | 0.00 |
| *E. coli* (ESBL) | 200 mg/mL | 15.81 | 18.70 | 23.00 |
|  | 100 mg/mL | 11.31 | 12.82 | 15.07 |
|  | 50 mg/mL | 5.69 | 3.90 | 2.70 |
|  | 25 mg/mL | 4.93 | 7.16 | 5.82 |
|  | Cipro 50 µg/mL | 26.41 | 26.16 | 25.44 |
|  | DMSO | 0.00 | 0.00 | 0.00 |
| *Klebsiella pneumoniae* | 200 mg/mL | 24.23 | 31.93 | 27.33 |
|  | 100 mg/mL | 11.26 | 13.47 | 16.76 |
|  | 50 mg/mL | 7.45 | 10.34 | 8.61 |
|  | 25 mg/mL | 1.69 | 1.03 | 0.58 |
|  | Cipro 50 µg/mL | 23.44 | 24.16 | 24.41 |
|  | DMSO | 0.00 | 0.00 | 0.00 |
| *K. pneumoniae* (ESBL) | 200 mg/mL | 13.81 | 15.67 | 18.43 |
|  | 100 mg/mL | 14.77 | 10.50 | 7.64 |
|  | 50 mg/mL | 3.72 | 7.32 | 5.16 |
|  | 25 mg/mL | 1.80 | 2.94 | 4.65 |
|  | Cipro 50 µg/mL | 28.44 | 29.16 | 29.41 |
|  | DMSO | 0.00 | 0.00 | 0.00 |
| *E. coli* ATCC 25922 | 200 mg/mL | 21.55 | 27.24 | 23.84 |
|  | 100 mg/mL | 17.65 | 14.48 | 12.36 |
|  | 50 mg/mL | 6.92 | 9.05 | 7.78 |
|  | 25 mg/mL | 2.57 | 3.28 | 4.32 |
|  | Cipro 50 µg/mL | 33.44 | 34.41 | 34.16 |
|  | DMSO | 0.00 | 0.00 | 0.00 |

**MIC Vernonia amygdalina (mg/mL)**

| Organism | Parameter | R1 | R2 | R3 |
| --- | --- | --- | --- | --- |
| *E. coli* ATCC 25922 | MIC | 12.50 | 12.50 | 12.50 |
| *S. typhi* | MIC | 25.00 | 25.00 | 25.00 |
| *S. aureus* | MIC | 25.00 | 25.00 | 25.00 |
| *E. coli* | MIC | 25.00 | 50.00 | 50.00 |
| *E. coli* (ESBL) | MIC | 12.50 | 12.50 | 12.50 |
| *K. pneumoniae* | MIC | 12.50 | 12.50 | 25.00 |
| *K. pneumoniae* (ESBL) | MIC | 12.50 | 12.50 | 12.50 |

**MBC Vernonia amygdalina (mg/mL)**

| Organism | Parameter | R1 | R2 | R3 |
| --- | --- | --- | --- | --- |
| *E. coli* ATCC 25922 | MBC | 25.00 | 25.00 | 25.00 |
| *S. typhi* | MBC | 50.00 | 50.00 | 100.00 |
| *S. aureus* | MBC | 50.00 | 50.00 | 50.00 |
| *E. coli* | MBC | 100.00 | 100.00 | 100.00 |
| *E. coli* (ESBL) | MBC | 25.00 | 25.00 | 50.00 |
| *K. pneumoniae* | MBC | 25.00 | 25.00 | 25.00 |
| *K. pneumoniae* (ESBL) | MBC | 25.00 | 25.00 | 25.00 |

**MIC Senna siamea (mg/mL)**

| Organism | Parameter | R1 | R2 | R3 |
| --- | --- | --- | --- | --- |
| *E. coli* ATCC 25922 | MIC | 6.25 | 6.25 | 6.25 |
| *S. typhi* | MIC | 12.50 | 12.50 | 12.50 |
| *S. aureus* | MIC | 12.50 | 12.50 | 25.00 |
| *E. coli* | MIC | 25.00 | 25.00 | 50.00 |
| *E. coli* (ESBL) | MIC | 12.50 | 12.50 | 12.50 |
| *K. pneumoniae* | MIC | 6.25 | 6.25 | 12.50 |
| *K. pneumoniae* (ESBL) | MIC | 12.50 | 12.50 | 12.50 |

**MBC of Senna siamea (mg/mL)**

| Organism | Parameter | R1 | R2 | R3 |
| --- | --- | --- | --- | --- |
| *E. coli* ATCC 25922 | MBC | 12.50 | 12.50 | 12.50 |
| *S. typhi* | MBC | 12.50 | 25.00 | 25.00 |
| *S. aureus* | MBC | 25.00 | 25.00 | 25.00 |
| *E. coli* | MBC | 50.00 | 50.00 | 50.00 |
| *E. coli* (ESBL) | MBC | 25.00 | 25.00 | 25.00 |
| *K. pneumoniae* | MBC | 6.25 | 12.50 | 12.50 |
| *K. pneumoniae* (ESBL) | MBC | 25.00 | 25.00 | 25.00 |

**Synergistic MIC of Combined Plant Extracts (mg/mL)**

| Organism | Concentration | R1 | R2 | R3 | Organism |
| --- | --- | --- | --- | --- | --- |
| *S. typhi* | 6.25 mg/mL | 6.25 | 6.25 | 6.25 | S. typhi |
| *S. aureus* | 6.25 mg/mL | 6.25 | 6.25 | 6.25 | S. aureus |
| *E. coli* | 6.25 mg/mL | 6.25 | 6.25 | 6.25 | E. coli |
| *E. coli* (ESBL) | 12.5 mg/mL | 12.50 | 12.50 | 12.50 | E. coli (ESBL) |
| *K. pneumoniae* | 6.25 mg/mL | 6.25 | 6.25 | 6.25 | K. pneumoniae |
| *K. pneumoniae* (ESBL) | 6.25 mg/mL | 6.25 | 6.25 | 6.25 | K. pneumoniae (ESBL) |

**Ciprofloxacin + *Vernonia amygdalina* Synergistic MIC (µg/mL)**

| Organism | Concentration | R1 | R2 | R3 |
| --- | --- | --- | --- | --- |
| *S. typhi* | 6.25 µg/mL | 6.25 | 6.25 | 6.25 |
| *S. aureus* | 6.25 µg/mL | 6.25 | 6.25 | 6.25 |
| *E. coli* | 6.25 µg/mL | 6.25 | 6.25 | 6.25 |
| *E. coli* (ESBL) | 6.25 µg/mL | 6.25 | 6.25 | 6.25 |
| *K. pneumoniae* | 20 µg/mL | 12.50 | 25.00 | 25.00 |
| *K. pneumoniae* (ESBL) | 6.25 µg/mL | 6.25 | 6.25 | 6.25 |

**Ciprofloxacin + *Senna siamea* Synergistic MIC (µg/mL)**

| Organism | Concentration | R1 | R2 | R3 |
| --- | --- | --- | --- | --- |
| *S. typhi* | 0.391 µg/mL | 0.391 | 0.391 | 0.391 |
| *S. aureus* | 0.391 µg/mL | 0.391 | 0.391 | 0.391 |
| *E. coli* | 3.125 µg/mL | 3.125 | 3.125 | 3.125 |
| *E. coli* (ESBL) | 0.0977 µg/mL | 0.0977 | 0.0977 | 0.0977 |
| *K. pneumoniae* | 10 µg/mL | 6.25 | 12.50 | 12.50 |
| *K. pneumoniae* (ESBL) | 25 µg/mL | 25.00 | 25.00 | 25.00 |
