# Supplementary material for: Antimicrobial and anti-biofilm activities of Vernonia amygdalina and Senna siamea leaf extracts against pathogenic bacteria
Source: PLoS One. 2026 Oct 1;21(10):e0358840. doi: 10.1371/journal.pone.0358840 (PMC13630245; doi:10.1371/journal.pone.0358840)
Supplement: Peer review history

# Peer review: Decision Letter 0

**DOI:** 10.1371/journal.pone.0358840.r001

**Authors:** Christian Agyare

16 Feb 2026

Dear Dr. Boampong,

Thank you for submitting your manuscript to PLOS ONE. After careful consideration, we feel that it has merit but does not fully meet PLOS ONE’s publication criteria as it currently stands. Therefore, we invite you to submit a revised version of the manuscript that addresses the points raised during the review process.

Please submit your revised manuscript by Apr 02 2026 11:59PM. If you will need more time than this to complete your revisions, please reply to this message or contact the journal office at plosone@plos.org. When you're ready to submit your revision, log on to https://www.editorialmanager.com/pone/ and select the 'Submissions Needing Revision' folder to locate your manuscript file.

- A letter that responds to each point raised by the academic editor and reviewer(s). You should upload this letter as a separate file labeled 'Response to Reviewers'.
- A marked-up copy of your manuscript that highlights changes made to the original version. You should upload this as a separate file labeled 'Revised Manuscript with Track Changes'.
- An unmarked version of your revised paper without tracked changes. You should upload this as a separate file labeled 'Manuscript'.

If you would like to make changes to your financial disclosure, please include your updated statement in your cover letter. Guidelines for resubmitting your figure files are available below the reviewer comments at the end of this letter.

If applicable, we recommend that you deposit your laboratory protocols in protocols.io to enhance the reproducibility of your results. Protocols.io assigns your protocol its own identifier (DOI) so that it can be cited independently in the future. For instructions see: https://journals.plos.org/plosone/s/submission-guidelines#loc-laboratory-protocols. Additionally, PLOS ONE offers an option for publishing peer-reviewed Lab Protocol articles, which describe protocols hosted on protocols.io. Read more information on sharing protocols at https://plos.org/protocols?utm_medium=editorial-email&utm_source=authorletters&utm_campaign=protocols.

We look forward to receiving your revised manuscript.

Kind regards,

Christian Agyare, PhD

Academic Editor

PLOS One

Journal Requirements:

When submitting your revision, we need you to address these additional requirements.

1. Please ensure that your manuscript meets PLOS ONE's style requirements, including those for file naming. The PLOS ONE style templates can be found at

https://journals.plos.org/plosone/s/file?id=wjVg/PLOSOne_formatting_sample_main_body.pdf and

https://journals.plos.org/plosone/s/file?id=ba62/PLOSOne_formatting_sample_title_authors_affiliations.pdf.

2. In your Methods section, please provide additional information regarding the permits you obtained for the work. Please ensure you have included the full name of the authority that approved the field site access and, if no permits were required, a brief statement explaining why.

3. We note that your Data Availability Statement is currently as follows: [All relevant data are within the manuscript and its Supporting Information files]

Please confirm at this time whether or not your submission contains all raw data required to replicate the results of your study. Authors must share the “minimal data set” for their submission. PLOS defines the minimal data set to consist of the data required to replicate all study findings reported in the article, as well as related metadata and methods (https://journals.plos.org/plosone/s/data-availability#loc-minimal-data-set-definition).

For example, authors should submit the following data:

- The values behind the means, standard deviations and other measures reported;

- The values used to build graphs;

- The points extracted from images for analysis.

Authors do not need to submit their entire data set if only a portion of the data was used in the reported study.

If your submission does not contain these data, please either upload them as Supporting Information files or deposit them to a stable, public repository and provide us with the relevant URLs, DOIs, or accession numbers. For a list of recommended repositories, please see https://journals.plos.org/plosone/s/recommended-repositories.

If there are ethical or legal restrictions on sharing a de-identified data set, please explain them in detail (e.g., data contain potentially sensitive information, data are owned by a third-party organization, etc.) and who has imposed them (e.g., an ethics committee). Please also provide contact information for a data access committee, ethics committee, or other institutional body to which data requests may be sent. If data are owned by a third party, please indicate how others may request data access.

4. If the reviewer comments include a recommendation to cite specific previously published works, please review and evaluate these publications to determine whether they are relevant and should be cited. There is no requirement to cite these works unless the editor has indicated otherwise.

Additional Editor Comments (if provided):

Comments from Reviewer 1

1. Authors should justify the novelty of their work. The antimicrobial and antibiofilm activities of the plants have been reported. https://journaljocamr.com/index.php/JOCAMR/article/view/242

https://doi.org/10.58578/ajmsphr.v2i3.7038

https://doi.org/10.1101/2025.05.22.655479

2. Page 3 line 61; authors should clarify the statement ‘Biofilms are organized bacterial communities that are secreted by the bacteria themselves in an extracellular matrix’.

3. Provide adequate information on the two plants

4. The justification for the use of the 2 plants is weak and therefore, author should give a strong justification for the use of the plants

5. These plants have been reported to have antimicrobial activity, but no statement is on that in the introduction

6. The introduction suggests focussing on ESBL organisms, however there are other non-ESBL producing bacteria were employed in the study as well, authors should provide clarity

7. Are the organisms clinical or typed strains?

8. Authors should indicate the cork borer size used

9. Authors should explain why only one Gram-positive organism was used

10. Authors should indicate the extractive yield

11. How was the extract sterilized?

12. At what temperature was rotary evaporator set?

13. Page 5, line 100 -102; the information provided is not clear. Authors should revise to bring clarity

14. A summary table should be provided to indicate the specific tests performed for both the qualitative and quantitative identification of the stated phytochemicals

15. Line 113 how was the air-drying done

16. Line 115 change, was used to treat each well to was transferred into the appropriate well

17. Line 124 authors should state the concentrations of the extract used, volume of nutrient broth, and authors should indicate controls

18. Why did authors allow the organism to attach for 4 h? biofilm formation assay should be independent and not attached to inhibition assay

19. Line 169 were the wells allowed to dry before the CV was added?

20. Authors should indicate whether the solubilised CV were transferred into a separate 96 well plate before reading

21. Line 174: authors should clarify if it is OD of treated or OD of sample

22. Table 1: authors should check the key

23. Table 1: Values represent mean ± SD (n = 3). ND indicates not detected, should be added to key below the table

24. Tables 2 and 3: no standard deviation values for cipro

25. Tables 4 and 5: If MIC was done in 2-fold serial dilution how come authors are getting those values?

26. Line 256: authors should clarify how the extract promoted the formation of biofilm

27. Table 7: Values represent the percentage inhibition of biofilm formation at different concentrations (mg/mL). Negative values indicate biofilm promotion; authors should bring it below the table. They should do same for all

28. Line 288, authors should add the name of the author. E.g. Sampson et al. [22].

29. Biofilm inhibition assays use sub-MICs of extract. The authors should clarify why the used concentrations range from 25 to 100 mg/mL which is within the MIC range of the extracts. Hence, it is not possible to conclude that the activity may be due to biofilm inhibition.

Comments from Reviewer 2

The paper is a simple and straight forward paper, which is not too novel but very important in the era of antimicrobial resistance. The authors could have looked at more plant extracts than just the two they looked at in the discussion some of the statements are not complete. for instance on page 19, line 288, the authors wrote These findings align with [22],.... instead of writing 'these findings align with Ali et al [22]. There are quite a number of them in the discussion which need to be corrected.

Comments from Reviewer 3

Generally clear writing with logical organization. Appropriate use of tables for data presentation. Discussion adequately contextualizes findings within existing literature. Please address the following.

- Line 346-347- The combination of plant extracts with ciprofloxacin exhibited a greater synergistic effect as well. you did not test any extract-ciprofloxacin combinations. The synergy testing (Lines 132-157, Table 6) (Lines 116, 209, 221). The actual finding was synergy between the two extracts against E.coli only, Table 6, Line 251.

- Lines 30-31- Senna siamea, there was a significant difference only among the organisms, p = 0.9161 (appears only in abstract). The Results section (Lines 216-218). The discrepancy between p = 0.9161 (abstract) and p = 0.231 (results) must also be explained.

- Not a statistician, but ANOVA results for the two extracts show incompatible degrees of freedom structures. The denominator df differ a lot (48 vs. 15). Explain. Also, no details on interaction data? Include all the stats as supplementary files.

- Add dose-response figures. At least one figure in the whole MS.

- Abstract (Lines 27-28): S. siamea MIC range reported as 8.33 ± 3.61-33.33 ± 14.43 mg/mL, Table 5 (Line 241): Shows MIC for E. coli ATCC 25922 as 6.25 ± 0.00 mg/mL

- Line 100 - sterilized extract- how was this done?

- Report extraction yield, describe the sterilization procedure, and detail how working concentrations were prepared from the dried extract.

- Lines 84-89- can you add more details

- Check the GPS coordinates appear reversed (Line 86-87)

- Lines 158-176- This experimental design tests biofilm development inhibition and not biofilm formation prevention. The terminology throughout the manuscript should be streamlined. It mixes these distinct mechanisms. Pre-formed (mature) biofilm disruption/eradication was not tested.

- Incomplete MIC/MBC Methodology (Lines 121-131)- full details, aerobic, inoclum density etc.

- Table 1, Lines 193-197- The quantitative values are reported in mg/g, mg per gram of dried extract or mg per gram of original plant material. Need to be revised in the MS.

- Why no positive controls to validate the assay system.

- Discussion more of Biofilm Promotion (Lines 257, 327-331). Sub-inhibitory concentrations promoting biofilm formation could have adverse therapeutic implications.

- Soften the language (e.g., "To our knowledge, this is among the first...").

- include Strain Information for Clinical Isolates- Lines 78-79

- Lines 204-206, 216-218, provide ANOVA tables as supplementary material

- Post-hoc test results not shown, Line 183 mentions LSD, Report pairwise comparisons for significant effects, as supplementary material

- Specify whether n=3 represents independent experiments (biological) or within-experiment replicates (technical)

- Ciprofloxacin values lack SD (reported as single values)

- Tables 7 & 8: Standard deviations not provided for biofilm inhibition percentages despite triplicate experiments

- Reference 31: What? business branding text (The future of purpose-driven branding) ChatGPT?

- Reference 9 and 28

[Note: HTML markup is below. Please do not edit.]

Reviewers' comments:

Reviewer's Responses to Questions

**Comments to the Author**

1. Is the manuscript technically sound, and do the data support the conclusions?

Reviewer #1: Partly

Reviewer #2: Yes

Reviewer #3: No

**********

2. Has the statistical analysis been performed appropriately and rigorously?

Reviewer #1: Yes

Reviewer #2: Yes

Reviewer #3: No

**********

3. Have the authors made all data underlying the findings in their manuscript fully available?

The PLOS Data policy

Reviewer #1: Yes

Reviewer #2: No

Reviewer #3: No

**********

4. Is the manuscript presented in an intelligible fashion and written in standard English?

Reviewer #1: Yes

Reviewer #2: Yes

Reviewer #3: Yes

**********

Reviewer #1: 1. Authors should justify the novelty of their work. The antimicrobial and antibiofilm activities of the plants have been reported. https://journaljocamr.com/index.php/JOCAMR/article/view/242

https://doi.org/10.58578/ajmsphr.v2i3.7038

https://doi.org/10.1101/2025.05.22.655479

2. Page 3 line 61; authors should clarify the statement ‘Biofilms are organized bacterial communities that are secreted by the bacteria themselves in an extracellular matrix’.

3. Provide adequate information on the two plants

4. The justification for the use of the 2 plants is weak and therefore, author should give a strong justification for the use of the plants

5. These plants have been reported to have antimicrobial activity, but no statement is on that in the introduction

6. The introduction suggests focussing on ESBL organisms, however there are other non-ESBL producing bacteria were employed in the study as well, authors should provide clarity

7. Are the organisms clinical or typed strains?

8. Authors should indicate the cork borer size used

9. Authors should explain why only one Gram-positive organism was used

10. Authors should indicate the extractive yield

11. How was the extract sterilized?

12. At what temperature was rotary evaporator set?

13. Page 5, line 100 -102; the information provided is not clear. Authors should revise to bring clarity

14. A summary table should be provided to indicate the specific tests performed for both the qualitative and quantitative identification of the stated phytochemicals

15. Line 113 how was the air-drying done

16. Line 115 change, was used to treat each well to was transferred into the appropriate well

17. Line 124 authors should state the concentrations of the extract used, volume of nutrient broth, and authors should indicate controls

18. Why did authors allow the organism to attach for 4 h? biofilm formation assay should be independent and not attached to inhibition assay

19. Line 169 were the wells allowed to dry before the CV was added?

20. Authors should indicate whether the solubilised CV were transferred into a separate 96 well plate before reading

21. Line 174: authors should clarify if it is OD of treated or OD of sample

22. Table 1: authors should check the key

23. Table 1: Values represent mean ± SD (n = 3). ND indicates not detected, should be added to key below the table

24. Tables 2 and 3: no standard deviation values for cipro

25. Tables 4 and 5: If MIC was done in 2-fold serial dilution how come authors are getting those values?

26. Line 256: authors should clarify how the extract promoted the formation of biofilm

27. Table 7: Values represent the percentage inhibition of biofilm formation at different concentrations (mg/mL). Negative values indicate biofilm promotion; authors should bring it below the table. They should do same for all

28. Line 288, authors should add the name of the author. E.g. Sampson et al. [22].

29. Biofilm inhibition assays use sub-MICs of extract. The authors should clarify why the used concentrations range from 25 to 100 mg/mL which is within the MIC range of the extracts. Hence, it is not possible to conclude that the activity may be due to biofilm inhibition.

Reviewer #2: The paper is a simple and straight forward paper, which is not too novel but very important in the era of antimicrobial resistance. The authors could have looked at more plant extracts than just the two they looked at. in the discussion some of the statements are not complete. for instance on page 19, line 288, the authors wrote These findings align with [22],.... instead of writing 'these findings align with Ali et al [22]. There are quite a number of them in the discussion which need to be corrected.

Reviewer #3: Generally clear writing with logical organization. Appropriate use of tables for data presentation. Discussion adequately contextualizes findings within existing literature. Please address the following.

- Line 346-347- The combination of plant extracts with ciprofloxacin exhibited a greater synergistic effect as well. you did not test any extract-ciprofloxacin combinations. The synergy testing (Lines 132-157, Table 6) (Lines 116, 209, 221). The actual finding was synergy between the two extracts against E.coli only, Table 6, Line 251.

- Lines 30-31- Senna siamea, there was a significant difference only among the organisms, p = 0.9161 (appears only in abstract). The Results section (Lines 216-218). The discrepancy between p = 0.9161 (abstract) and p = 0.231 (results) must also be explained.

- Not a statistician, but ANOVA results for the two extracts show incompatible degrees of freedom structures. The denominator df differ a lot (48 vs. 15). Explain. Also, no details on interaction data? Include all the stats as supplementary files.

- Add dose-response figures. At least one figure in the whole MS.

- Abstract (Lines 27-28): S. siamea MIC range reported as 8.33 ± 3.61-33.33 ± 14.43 mg/mL, Table 5 (Line 241): Shows MIC for E. coli ATCC 25922 as 6.25 ± 0.00 mg/mL

- Line 100 - sterilized extract- how was this done?

- Report extraction yield, describe the sterilization procedure, and detail how working concentrations were prepared from the dried extract.

- Lines 84-89- can you add more details

- Check the GPS coordinates appear reversed (Line 86-87)

- Lines 158-176- This experimental design tests biofilm development inhibition and not biofilm formation prevention. The terminology throughout the manuscript should be streamlined. It mixes these distinct mechanisms. Pre-formed (mature) biofilm disruption/eradication was not tested.

- Incomplete MIC/MBC Methodology (Lines 121-131)- full details, aerobic, inoclum density etc.

- Table 1, Lines 193-197- The quantitative values are reported in mg/g, mg per gram of dried extract or mg per gram of original plant material. Need to be revised in the MS.

- Why no positive controls to validate the assay system.

- Discussion more of Biofilm Promotion (Lines 257, 327-331). Sub-inhibitory concentrations promoting biofilm formation could have adverse therapeutic implications.

- Soften the language (e.g., "To our knowledge, this is among the first...").

- include Strain Information for Clinical Isolates- Lines 78-79

- Lines 204-206, 216-218, provide ANOVA tables as supplementary material

- Post-hoc test results not shown, Line 183 mentions LSD, Report pairwise comparisons for significant effects, as supplementary material

- Specify whether n=3 represents independent experiments (biological) or within-experiment replicates (technical)

- Ciprofloxacin values lack SD (reported as single values)

- Tables 7 & 8: Standard deviations not provided for biofilm inhibition percentages despite triplicate experiments

- Reference 31: What? business branding text (The future of purpose-driven branding) ChatGPT?

- Reference 9 and 28

**********

what does this mean?). If published, this will include your full peer review and any attached files.

If you choose “no”, your identity will remain anonymous but your review may still be made public.

**Do you want your identity to be public for this peer review?**  For information about this choice, including consent withdrawal, please see our Privacy Policy

Reviewer #1: No

Reviewer #2: No

Reviewer #3: No

**********

[NOTE: If reviewer comments were submitted as an attachment file, they will be attached to this email and accessible via the submission site. Please log into your account, locate the manuscript record, and check for the action link "View Attachments". If this link does not appear, there are no attachment files.]

To ensure your figures meet our technical requirements, please review our figure guidelines: https://journals.plos.org/plosone/s/figures

You may also use PLOS’s free figure tool, NAAS, to help you prepare publication quality figures: https://journals.plos.org/plosone/s/figures#loc-tools-for-figure-preparation.

NAAS will assess whether your figures meet our technical requirements by comparing each figure against our figure specifications.

Attachment Submitted filename: Comments from Reviewers.pdf

# Author comment: Author response to Decision Letter 1

**DOI:** 10.1371/journal.pone.0358840.r002

30 Apr 2026

Reviewer’s Comment / Issue Author’s Response

Novelty of the study is not justified. Addressed. The introduction was revised to clearly explain the relevance of the study. The novelty claim was moderated and supported with existing literature. The study aim was clarified to include both ESBL and non-ESBL organisms.

Weak justification for plant selection Addressed. Justification for selecting Vernonia amygdalina and Senna siamea was added based on ethnomedicinal use and reported antimicrobial properties.

Prior antimicrobial studies were not cited. Addressed. Relevant studies on antimicrobial and antibiofilm activity of both plants were incorporated.

The organism source is not clear. Addressed. Sources of isolates and strain details were clearly stated, including reference strains.

Lack of reference strain information Addressed. A reference strain such as E. coli ATCC 25922 was included.

Extraction procedure incomplete Addressed. Extraction yield, drying method, rotary evaporator temperature, and sterilization method were added.

Preparation of concentrations unclear Addressed. Stock and working solution preparation were clearly described.

The MIC method is not fully described. Addressed. Full broth microdilution method, inoculum density, and incubation conditions were added.

Controls not described Addressed. Positive and negative controls were clearly defined.

Biofilm assay unclear Addressed. Assay procedure was clarified. Study defined as early biofilm inhibition. Washing, drying, and OD measurement were explained.

Biofilm promotion is not explained. Addressed. Observed promotion at low concentrations was discussed scientifically in the results.

Synergy interpretation incorrect Addressed. Incorrect claims were removed. Conclusions revised to reflect actual findings.

Statistical inconsistencies Addressed. Data were reanalyzed. P-values corrected and made consistent across sections.

Missing ANOVA and post hoc tests Addressed. ANOVA tables and post hoc results were added as supplementary material.

Tables lack SD and clarity. Addressed. All tables updated to include mean ± SD. Legends and units clarified.

Phytochemical units unclear Addressed. Units were defined, and the table was revised accordingly.

No figures included Addressed. Dose-dependent curves and relevant figures were added.

Raw data not provided Addressed. Raw datasets and replicates were added as supplementary files.

Poor referencing and citation errors Addressed. All references were verified, corrected, and formatted. Missing citations were added.

Writing and formatting issues Addressed. The entire manuscript was edited for grammar, clarity, and consistency.

Attachment Submitted filename: Responses to Reviewer.docx

# Peer review: Decision Letter 1

**DOI:** 10.1371/journal.pone.0358840.r003

**Authors:** Muhammad Hussnain Siddique

10 Jul 2026

Dear Dr. Boampong,

Please submit your revised manuscript by Aug 24 2026 11:59PM. If you will need more time than this to complete your revisions, please reply to this message or contact the journal office at plosone@plos.org. When you're ready to submit your revision, log on to https://www.editorialmanager.com/pone/ and select the 'Submissions Needing Revision' folder to locate your manuscript file.

- A letter that responds to each point raised by the academic editor and reviewer(s). You should upload this letter as a separate file labeled 'Response to Reviewers'.
- A marked-up copy of your manuscript that highlights changes made to the original version. You should upload this as a separate file labeled 'Revised Manuscript with Track Changes'.
- An unmarked version of your revised paper without tracked changes. You should upload this as a separate file labeled 'Manuscript'.

If you would like to make changes to your financial disclosure, please include your updated statement in your cover letter. Guidelines for resubmitting your figure files are available below the reviewer comments at the end of this letter.

If applicable, we recommend that you deposit your laboratory protocols in protocols.io to enhance the reproducibility of your results. Protocols.io assigns your protocol its own identifier (DOI) so that it can be cited independently in the future. For instructions see: https://journals.plos.org/plosone/s/submission-guidelines#loc-laboratory-protocols. Additionally, PLOS ONE offers an option for publishing peer-reviewed Lab Protocol articles, which describe protocols hosted on protocols.io. Read more information on sharing protocols at https://plos.org/protocols?utm_medium=editorial-email&utm_source=authorletters&utm_campaign=protocols.

As the corresponding author, your ORCID iD is verified in the submission system and will appear in the published article. PLOS supports the use of ORCID, and we encourage all coauthors to register for an ORCID iD and use it as well. Please encourage your coauthors to verify their ORCID iD within the submission system before final acceptance, as unverified ORCID iDs will not appear in the published article. *Only*  the individual author can complete the verification step; PLOS staff *cannot*  verify ORCID iDs on behalf of authors.

We look forward to receiving your revised manuscript.

Kind regards,

Muhammad Hussnain Siddique

Academic Editor

PLOS One

Journal Requirements:

1. If the reviewer comments include a recommendation to cite specific previously published works, please review and evaluate these publications to determine whether they are relevant and should be cited. There is no requirement to cite these works unless the editor has indicated otherwise.

2. Please review your reference list to ensure that it is complete and correct. If you have cited papers that have been retracted, please include the rationale for doing so in the manuscript text, or remove these references and replace them with relevant current references. Any changes to the reference list should be mentioned in the rebuttal letter that accompanies your revised manuscript. If you need to cite a retracted article, indicate the article’s retracted status in the References list and also include a citation and full reference for the retraction notice.

[Note: HTML markup is below. Please do not edit.]

Reviewer's Responses to Questions

**Comments to the Author**

Reviewer #2: All comments have been addressed

Reviewer #3: (No Response)

**********

2. Is the manuscript technically sound, and do the data support the conclusions?

Reviewer #2: Yes

Reviewer #3: Partly

**********

3. Has the statistical analysis been performed appropriately and rigorously?

Reviewer #2: Yes

Reviewer #3: Yes

**********

4. Have the authors made all data underlying the findings in their manuscript fully available?

The PLOS Data policy

Reviewer #2: Yes

Reviewer #3: No

**********

5. Is the manuscript presented in an intelligible fashion and written in standard English?

Reviewer #2: Yes

Reviewer #3: Yes

**********

Reviewer #2: (No Response)

Reviewer #3: Thanks to the authors for addressing many points I raised earlier. These below still need work.

- Line 346-347: extract-plus-ciprofloxacin synergy was never tested; the only synergy was between the two extracts against E. coli (Table 6).

o Abstract fixed, but the Conclusion still makes the ciprofloxacin claim. Remove it.

- Lines 30-31: p = 0.9161 (abstract) vs p = 0.231 (results) must be explained.

o The MIC ANOVA is still in the abstract with no matching analysis anywhere. Provide it or delete it.

- Full ANOVA tables are now in the supplement, but Methods say LSD while every table report Tukey.

- Abstract MIC range 8.33 to 33.33 vs Table 5 showing 6.25.

o Text, Table 5, and Discussion now give different MICs. Reconcile

- clarify mg/g (per dried extract or per plant material).

- References 9 and 28 (you can use Zotero to get your referencing done properly). Even google scholar will do it.

**********

what does this mean?). If published, this will include your full peer review and any attached files.

If you choose “no”, your identity will remain anonymous but your review may still be made public.

**Do you want your identity to be public for this peer review?**  For information about this choice, including consent withdrawal, please see our Privacy Policy

Reviewer #2: No

Reviewer #3: **Yes:** abhinandan kumar

**********

[NOTE: If reviewer comments were submitted as an attachment file, they will be attached to this email and accessible via the submission site. Please log into your account, locate the manuscript record, and check for the action link "View Attachments". If this link does not appear, there are no attachment files.]

To ensure your figures meet our technical requirements, please review our figure guidelines: https://journals.plos.org/plosone/s/figures

You may also use PLOS’s free figure tool, NAAS, to help you prepare publication quality figures: https://journals.plos.org/plosone/s/figures#loc-tools-for-figure-preparation.

NAAS will assess whether your figures meet our technical requirements by comparing each figure against our figure specifications.

# Author comment: Author response to Decision Letter 2

**DOI:** 10.1371/journal.pone.0358840.r004

24 Jul 2026

Response to Reviewers

Manuscript: Antimicrobial and Anti-Biofilm Activities of Vernonia amygdalina and Senna siamea Leaf Extracts against Pathogenic Bacteria

We thank Reviewer #3 for the careful re-review and for identifying the remaining inconsistencies. We have addressed each point below and revised the manuscript accordingly. A point-by-point response follows, indicating the specific location of each change in the revised manuscript.

Reviewer #2

No response was provided by Reviewer #2; no corresponding changes were required.

Reviewer #3

Reviewer Comment Author Response Location of Change

Reviewer #3 (Point 1): The extract-plus-ciprofloxacin synergy was never tested; the only synergy demonstrated was between the two plant extracts against E. coli (Table 6). The Abstract was corrected previously, but the Conclusion still made the ciprofloxacin claim. We thank the reviewer for catching this inconsistency. The Conclusion has been revised to remove the claim of extract-plus-ciprofloxacin synergy. It now states only that the combination of the two plant extracts exhibited a synergistic effect against E. coli, consistent with the FIC index data reported in Table 6. No claim regarding ciprofloxacin combination synergy remains anywhere in the manuscript. Conclusion section, paragraph 1

Reviewer #3 (Point 2): The MIC ANOVA reported in the Abstract (p = 0.9161) does not match the Results (p = 0.231), and no corresponding analysis appears anywhere else in the manuscript. Provide the analysis or delete it. We have removed the MIC ANOVA statement from the Abstract. MIC values are ordinal, two-fold dilution endpoints (e.g., 6.25, 12.5, 25, 50 mg/mL) rather than continuous measurements, and several test organisms in our data show zero variance across replicates (SD = 0.00). These properties mean the assumptions underlying ANOVA (continuous, normally distributed data with estimable within-group error) are not met, and we do not have grounds to report a valid inferential statistic for the MIC data. Consistent with common practice in antimicrobial susceptibility reporting, MIC and MBC values are now presented descriptively (mean +/- SD, or the most consistent value across replicates) in the Abstract, Results, and Discussion, without an accompanying ANOVA or p-value. Abstract

Reviewer #3 (Point 3): Full ANOVA tables are now in the Supplement, but the Methods state that a Least Significant Difference (LSD) post hoc test was used, while every table reports Tukey's test. This inconsistency has been corrected. The Statistical Analysis subsection of the Methods now states that post hoc comparisons were made using Tukey's Honestly Significant Difference (HSD) test, which matches the post hoc test reported in every ANOVA table in the manuscript and Supplement. Methods, Statistical Analysis subsection

Reviewer #3 (Point 4): The Abstract reports an MIC range of 8.33-33.33 mg/mL for Senna siamea, while Table 5 shows a value of 6.25 mg/mL. The text, Table 5, and Discussion give different MIC values; these need to be reconciled. The MIC values for Senna siamea have been reconciled across the entire manuscript. The Abstract, Results narrative, and Discussion now all report the same range, 6.25 to 25.00 mg/mL, matching the values presented in Table 5 exactly. No discrepant MIC figures for Senna siamea remain in the manuscript. Abstract; Results narrative preceding Table 5; Discussion

Reviewer #3 (Point 5): Please clarify whether the phytochemical concentrations (mg/g) are expressed per dried extract or per plant material. This has been clarified. A statement has been added specifying that quantitative phytochemical values are expressed as mg per gram of the dried crude extract (not per unit mass of raw plant material). The units in the Table 1 caption and column headers, as well as in the accompanying narrative text, have been updated to explicitly state "mg/g of dried extract" throughout. Results, Phytochemical Composition subsection; Table 1 caption and column headers

Reviewer #3 (Point 6): References 9 and 28 are incomplete/improperly formatted; the reviewer suggested using Zotero or Google Scholar to correct the citations. Both references have been corrected using the original source records. Reference 9 (Singh, Lalbiaktluangi, Zomuansangi, Srivastava, Yadav, and Gupta, 2024) now includes the full author list, book chapter title, editors/volume title, publisher, page range, and DOI. Reference 28 (Hassan, Nasiru, Malut, Abdulkadir, and Ali, 2015) now includes the full author list, correct journal name (Journal of Pharmacognosy and Phytotherapy), volume, issue, page range, and DOI. Reference list, items 9 and 28

All changes described above are also highlighted in yellow in the accompanying Manuscript_Corrected_Version_Highlighted.docx file for the reviewer's convenience.

Attachment Submitted filename: Response to Reviewers.docx

# Peer review: Decision Letter 2

**DOI:** 10.1371/journal.pone.0358840.r005

**Authors:** Muhammad Hussnain Siddique

1 Sep 2026

Antimicrobial and Anti-Biofilm Activities of Vernonia amygdalina and Senna siamea Leaf Extracts Against Pathogenic Bacteria

PONE-D-25-66059R2

Dear Dr. Boampong,

We’re pleased to inform you that your manuscript has been judged scientifically suitable for publication and will be formally accepted for publication once it meets all outstanding technical requirements.

Within one week, you’ll receive an e-mail detailing the required amendments. When these have been addressed, you’ll receive a formal acceptance letter and your manuscript will be scheduled for publication.

An invoice will be generated when your article is formally accepted. Please note, if your institution has a publishing partnership with PLOS and your article meets the relevant criteria, all or part of your publication costs will be covered. Please make sure your user information is up-to-date by logging into Editorial Manager at Editorial Manager® and clicking the ‘Update My Information' link at the top of the page. For questions related to billing, please contact billing support.

If your institution or institutions have a press office, please notify them about your upcoming paper to help maximize its impact. If they’ll be preparing press materials, please inform our press team as soon as possible -- no later than 48 hours after receiving the formal acceptance. Your manuscript will remain under strict press embargo until 2 pm Eastern Time on the date of publication. For more information, please contact onepress@plos.org.

Kind regards,

Muhammad Hussnain Siddique

Academic Editor

PLOS One

Additional Editor Comments (optional):

Reviewers' comments:

Reviewer's Responses to Questions

**Comments to the Author**

Reviewer #3: All comments have been addressed

**********

2. Is the manuscript technically sound, and do the data support the conclusions?

Reviewer #3: Yes

**********

3. Has the statistical analysis been performed appropriately and rigorously?

Reviewer #3: Yes

**********

4. Have the authors made all data underlying the findings in their manuscript fully available?

The PLOS Data policy

Reviewer #3: Yes

**********

5. Is the manuscript presented in an intelligible fashion and written in standard English?

Reviewer #3: Yes

**********

Reviewer #3: The authors have addressed the substantive points and the revision is much improved. I am satisfied with the responses and I recommend acceptance.

**********

what does this mean?). If published, this will include your full peer review and any attached files.

If you choose “no”, your identity will remain anonymous but your review may still be made public.

**Do you want your identity to be public for this peer review?**  For information about this choice, including consent withdrawal, please see our Privacy Policy

Reviewer #3: No

**********

# Editor report: Acceptance letter

**DOI:** 10.1371/journal.pone.0358840.r006

**Authors:** Muhammad Hussnain Siddique

PONE-D-25-66059R2

PLOS One

Dear Dr. Boampong,

I'm pleased to inform you that your manuscript has been deemed suitable for publication in PLOS One. Congratulations! Your manuscript is now being handed over to our production team.

At this stage, our production department will prepare your paper for publication. This includes ensuring the following:

* All references, tables, and figures are properly cited

* All relevant supporting information is included in the manuscript submission,

* There are no issues that prevent the paper from being properly typeset

You will receive further instructions from the production team, including instructions on how to review your proof when it is ready. Please keep in mind that we are working through a large volume of accepted articles, so please give us a few days to review your paper and let you know the next and final steps.

Lastly, if your institution or institutions have a press office, please let them know about your upcoming paper now to help maximize its impact. If they'll be preparing press materials, please inform our press team within the next 48 hours. Your manuscript will remain under strict press embargo until 2 pm Eastern Time on the date of publication. For more information, please contact onepress@plos.org.

You will receive an invoice from PLOS for your publication fee after your manuscript has reached the completed accept phase. If you receive an email requesting payment before acceptance or for any other service, this may be a phishing scheme. Learn how to identify phishing emails and protect your accounts at https://explore.plos.org/phishing.

If we can help with anything else, please email us at customercare@plos.org.

Thank you for submitting your work to PLOS One and supporting open access.

Kind regards,

PLOS One Editorial Office Staff

on behalf of

Dr. Muhammad Hussnain Siddique

Academic Editor

PLOS One
